# Isolated Ni^2+^ Cations as the Active Centers for 1‑Butene Dimerization in Zeolites

**DOI:** 10.1021/jacsau.5c00461

**Published:** 2025-06-30

**Authors:** Laura Löbbert, Abelina Ellert, Mengjie Zhou, Ricardo Bermejo-Deval, Noelia Barrabes, Rachit Khare, Maricruz Sanchez-Sanchez, Johannes A. Lercher

**Affiliations:** † Department of Chemistry and Catalysis Research Center, 9184 Technical University of Munich, Garching 85748, Germany; ‡ Institute of Material Chemistry, Vienna University of Technology, Vienna A-1060, Austria; § Institute of Chemical Environmental and Bioscience Engineering, Vienna University of Technology, Vienna 1060, Austria; ∥ Institute for Integrated Catalysis, Pacific Northwest National Laboratory, Richland, Washington 99354, United States

**Keywords:** butene dimerization, Ni zeolites, isolated Ni sites, shape selectivity

## Abstract

We have identified isolated Ni^2+^ cations, ion-exchanged at the Al-pair sites, as the active centers for 1-butene dimerization under supercritical reaction conditions (*T* ≈ 433 K and *p*_butene_ ≈ 42.5 bar) on three different zeolite frameworks, viz., small-pore CHA, medium-pore MFI, and large-pore FAU. The linear octene selectivity, at low 1-butene conversions, decreased systematically with the size of the pore openings of the zeolites: CHA (∼50%) ≈ MFI (∼46%) > FAU (∼27%). The turnover frequency for 1-butene conversion, on the other hand, followed the order: FAU (∼19.2 mol_butene_ mol_Ni_^–1^ s^–1^) ≫ MFI (∼0.36 mol_butene_ mol_Ni_^–1^ s^–1^) ≈ CHA (∼0.33 mol_butene_ mol_Ni_^–1^ s^–1^). Butene dimerization proceeds via a Cossee–Arlman-type coordination-insertion mechanism on in situ generated Ni-butyl complexes as the active reaction centers. The differences in reactivity stem from a lower intrinsic activation energy for C–C coupling in FAU due to the more spacious environment of its supercages. The larger pores of FAU stabilize adsorbed 1-butene less than the pores of CHA and MFI frameworks but stabilize the bulkier C–C coupling transition state better than the latter two. These varying degrees of stabilization of reactant and transition states result in the almost two orders of magnitude higher dimerization activity in Ni-exchanged FAU zeolites compared to CHA or MFI.

## Graphical abstract

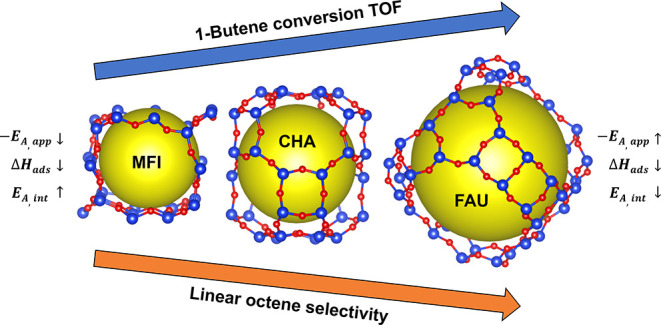

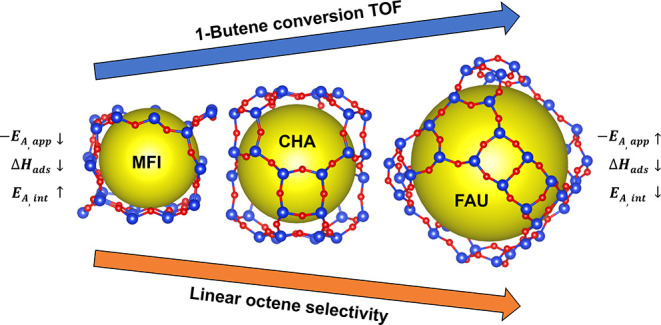

## Introduction

The formation of new carbon–carbon bonds via oligomerization is a promising approach toward synthesizing larger, more complex hydrocarbons from light olefins.^1–5^ Branched products, such as dimethylhexenes in the case of butene dimerization, serve as key intermediates in the production of gasoline additives. Linear products, on the other hand, find use as solvents, lubricants, and as comonomers in the manufacturing of linear low-density polyethylene.^6^ The linear dimers can also undergo hydroformylation followed by hydrogenation to produce phthalic esters, which are essential raw materials for manufacturing plasticizers.^6,7^ Given the industrial significance of these linear products, the development of highly active catalysts with superior selectivity for their production is essential.

Ni-based materials have been widely recognized as the most promising catalysts for alkene oligomerization owing to their high overall activity and high selectivity toward linear products.^8–12^ Consequently, these Ni-based catalysts have also seen commercial success, for example, in the homogeneous IFP Dimersol process^13^ and the heterogeneous OCTOL process developed by Evonik Industries.^1^ The OCTOL process employs a bifunctional Ni oxide catalyst supported on amorphous aluminosilicates (ASA) operating at 70–120 °C and 25–35 bar butene partial pressure and offers several advantages over the homogeneous process, including the ease of catalyst separation.^1,14^

The activity, selectivity, and deactivation behavior of the active sites are significantly impacted by the properties of the support material.^10^ Especially, the residual Brønsted acid sites (BAS) present in the support materials are detrimental to the linear dimer selectivity.^15^ BAS catalyze undesired side reactions such as skeletal isomerization and cracking, all of which result in a product composition that excludes the linear dimers.^16–18^ In addition to lowering the selectivity toward linear dimers, these BAS also catalyze the production of heavier hydrocarbons and coke precursors, which eventually caused pore blockage and accelerated catalyst deactivation.^19^

The local environment around the active site has also been shown to influence the catalyst activity and product distribution during alkene dimerization.^20^ For example, Mlinar et al. demonstrated that increasing the space around the active Ni cations in mesoporous ASA enhanced propene/alkene dimerization rates.^21,22^ The choice of support is, therefore, essential not only for stabilizing the active sites during catalyst activation^23,24^ but also for maintaining their stability during operation.^25^ As a result, a variety of support materials have been investigated for alkene dimerization reactions.^10^

Zeolites, with their ordered micro- and mesoporous channel systems, are particularly well-suited to investigate the impact of the local environment around the active Ni sites on both their activity and the selectivity toward linear products. In a previous work, we have demonstrated that Ni-containing LTA zeolites effectively catalyze dimerization of 1-butene under supercritical reaction conditions (at *T* ≈ 433 K and *p*_total_ ≈ 50 bar) while maintaining exceptionally high linear octene selectivity (almost 55% at low 1-butene conversions).^8^ This linear octene selectivity was substantially higher than those reported for Ni-ASA catalysts at similar conversions.^8,26^

Several potential active species have been proposed for alkene oligomerization reactions on Ni-based catalysts, such as Ni–H species in Ni-BEA and Ni-UiO-66,^27–29^ isolated Ni^2+^ cations in different Ni-zeolites,^8,24,28^ reduced Ni^1+^ cations in Ni-FAU zeolites,^30–32^ [Ni­(II)­OH]^+^ in Ni-MCM-41,^33,34^ NiOH in Ni-ASA,^9,25^ monomeric Ni sites and oxo complexes in Ni-UiO-66,^35^ and mobile Ni­(II) complexes in Ni-exchanged zeolites.^36^ The ambiguity with respect to the nature of the active sites, as well as the influence of their surrounding chemical environment, limits a deeper understanding of the alkene dimerization mechanism in these Ni-based catalysts.

The mechanism of dimerization on Ni-exchanged zeolites remains under discussion, and several mechanisms have been proposed.^37^ These include the Cossee–Arlman-type coordination insertion mechanism involving Ni-cationic or Ni-alkyl complexes,^8,36^ the metallacycle mechanism,^3,38^ the proton-transfer mechanism,^10,39^ and the Ni^1+^/Ni^2+^ redox shuttle mechanism,^25^ to name a few. In some mechanisms, additionally, Brønsted acidic protons have been suggested to actively participate in the reaction.^25,40,41^ In our previous work, using kinetic studies combined with spectroscopy, we proposed that 1-butene dimerization on the isolated Ni^2+^ cations in Ni–Ca-LTA zeolites proceeds via a Cossee–Arlman-type mechanism under supercritical reaction conditions. The reaction was postulated to proceed on the in situ generated Ni-butyl complexes.^8^

This work aims to identify the active Ni species (for 1-butene dimerization) in Ni-exchanged zeolites and the effect of the local environment on their activity and selectivity under supercritical reaction conditions (*T* ≈ 433 K and *p*_butene_ ≈ 42.5 bar). For this, we have investigated three different zeolite frameworks: small-pore CHA, medium-pore MFI, and large-pore FAU. Zeolite samples with similar Si/Al ratios were selected to ensure comparable acid site concentration and ion exchangeability. X-ray absorption spectroscopy (XAS) and infrared (IR) spectroscopy identified the active sites in these Ni-exchanged zeolites as isolated Ni^2+^ cations ion-exchanged at Al pair locations. The variation in pore opening, pore size, and interconnectivity in these zeolite frameworks provides valuable insight into how spatial constraints around the active site impact the activity and selectivity of Ni^2+^ cations for 1-butene dimerization.

## Results and Discussion

### 1-Butene Dimerization on Ni-NaCHA Zeolites

Let us first look at 1-butene dimerization on Ni-exchanged small-pore CHA zeolites under supercritical reaction conditions. Table 1 summarizes the total Al content and Si/Al ratios, estimated from atomic absorption spectroscopy (AAS), and the Al-pair concentrations, estimated from Co^2+^ ion exchange, in three different CHA zeolite samples, showing low, medium, and high concentration of Al pairs. These parent CHA zeolites are hereon referred to as (i) CHA_L_ (∼33% Al pairs), (ii) CHA_M_ (∼47% Al pairs), and (iii) CHA_H_ (∼95% Al pairs). These parent zeolites were first ion-exchanged with Na^+^ and then with Ni^2+^ to reduce the BAS concentration. The Ni and Na contents in the synthesized Ni-NaCHA zeolites are summarized in Supporting Information Tables S1, S2 and S3.

**Table 1:** Si/Al Ratios, Total Al Content, and Al-Pair Concentration in the Parent HCHA, HMFI, and HFAU Zeolite Samples

zeolite	Si/Al	Al content/μmol_Al_ g_zeolite_^–1^	Al pair content^a^/μmol_Al‑pair_·g_zeolite_^–1^
HCHA_L_	∼15	∼900	∼150 (0.33)
HCHA_M_	∼12	∼1200	∼280 (0.47)
HCHA_H_	∼11	∼1395	∼690 (0.95)
HMFI	∼15	∼970	∼270 (0.56)
HFAU	∼15	∼1000	∼310 (0.62)

a Estimated from Co^2+^ ion exchange. In parentheses, the fraction of Al that exists as Al pairs.

X-ray diffraction (XRD), N_2_ physisorption, and scanning electron microscopy (SEM) (see Supporting Information Section S2) confirmed that the incorporation of Ni did not compromise the crystallinity or the morphology of the parent CHA zeolites. Furthermore, IR spectroscopy did not show the presence of significant amount of BAS in the synthesized Ni-NaCHA zeolite samples.

Figure 1 shows the 1-butene consumption rates (*r*_butene_) on Ni-NaCHA zeolites as a function of their Ni content under differential (<10%) conversion conditions. Under these reaction conditions, *r*_butene_, when normalized to the catalyst mass, increased linearly with increasing Ni content until the Ni loading reached the concentration of Al pairs for each series of Ni-NaCHA zeolites (see dashed lines; Figure 1). This linear trend suggests that the dimerization activity in Ni-NaCHA zeolites is directly linked to isolated Ni^2+^ cations, preferentially exchanged at the Al-pair sites in these zeolites.

**Figure 1:**
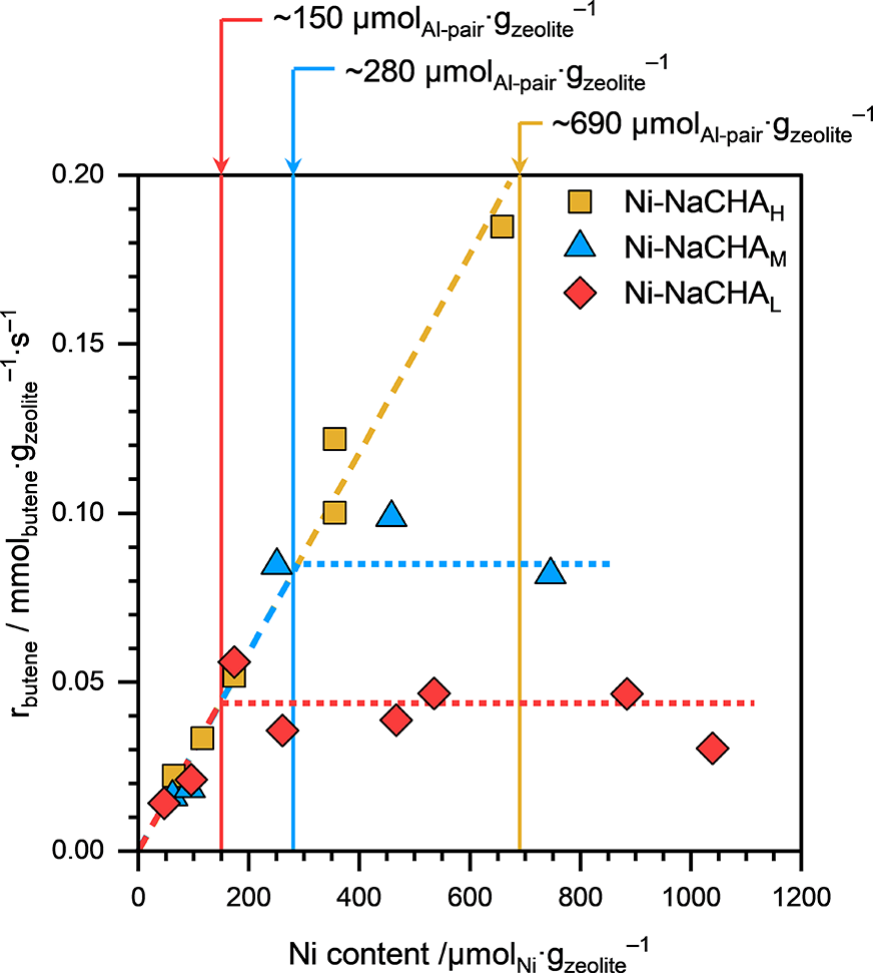
Rate of 1-butene consumption (*r*_butene_), normalized to the catalyst mass, on Ni-NaCHA zeolite samples with different Al-pair concentrations and Ni loadings, as a function of their Ni content. Reaction conditions: *T* ≈ 433 K, *p*_total_ ≈ 50 bar (15% isobutane and 85% 1-butene), space-velocity: 0.03–2.5 mmol_butene_·g_zeolite_·s^–1^. All reactions were performed under differential (<10%) conversion conditions. The solid lines represent the Al-pair concentrations in the parent HCHA_L_ (red), HCHA_M_ (blue), and HCHA_H_ (yellow) zeolites.

From the slope of the *r*_butene_ versus Ni content plots (dashed lines; Figure 1), the 1-butene dimerization turnover frequency (TOF_butene_) on the isolated Ni^2+^ sites in the CHA framework was estimated to be 0.33 ± 0.02 mol_butene_·mol_Ni_^–1^·s^–1^. We also performed 1-butene dimerization, under similar supercritical reaction conditions, on the parent HCHA_L_ zeolite sample and from these experiments, the TOF_butene_ on the BAS in the CHA framework was estimated to be ∼0.074 mol_butene_·mol_BAS_^–1^·s^–1^, significantly lower than that estimated for Ni^2+^ sites in Ni-NaCHA zeolites.

Once Ni loading exceeded the Al-pair exchange limit, *r*_butene_ (when normalized to the catalyst mass) did not increase further and remained almost constant with an increasing Ni content for each series of Ni-NaCHA zeolites (dotted lines; Figure 1). This behavior suggests that with all available Al-pair locations occupied by isolated Ni^2+^ cations, excess Ni was either exchanged at the isolated Al sites (for example, as [Ni­(OH)]^+^ cations) or aggregated as neutral NiO nanoclusters within the zeolite micropores or on the external zeolite surface. In either case, these additional sites are (i) inactive for 1-butene dimerization under the investigated reaction conditions and (ii) were not formed at the expense of active Ni sites.

### 1-Butene Dimerization on Ni-NaMFI Zeolites

Next, we investigated 1-butene dimerization on Ni-exchanged medium-pore MFI zeolites under supercritical reaction conditions. The total Al content and Si/Al ratio (estimated from AAS) and Al pair concentration (estimated from Co^2+^ ion exchange) in the parent HMFI zeolite are reported in Table 1. Structural characterization of the synthesized Ni-NaMFI zeolites (see Supporting Information Section S3) confirmed that the zeolite framework remained intact during the entire ion-exchange procedure. The Ni and Na contents, estimated from AAS, and the BAS and LAS concentrations, determined from IR spectroscopy of adsorbed pyridine, in the synthesized Ni-NaMFI zeolite samples are summarized in Supporting Information Table S4. Notably, despite prior ion exchange with Na^+^, all Ni-NaMFI zeolite samples retained a small amount of residual BAS.

Figure 2 presents 1-butene consumption rates normalized to the catalyst mass (*r*_butene_) on Ni-NaMFI zeolites as a function of their Ni contents at differential (<10%) conversion conditions. Similar to the Ni-NaCHA zeolite samples, *r*_butene_, when normalized to the catalyst mass, increased almost linearly with increasing Ni content (see dashed line; Figure 2). The linear trend continued until the Ni loading reached the concentration of the Al pair in the parent MFI zeolite (i.e., ∼270 μmol_Al‑pair_·g_zeolite_^–1^). Similar to the case of Ni-NaCHA zeolite samples, this trend suggests that the 1-butene dimerization activity of Ni-NaMFI zeolites is likely linked to isolated Ni^2+^ cations exchanged at the Al-pair sites within the MFI framework.

**Figure 2:**
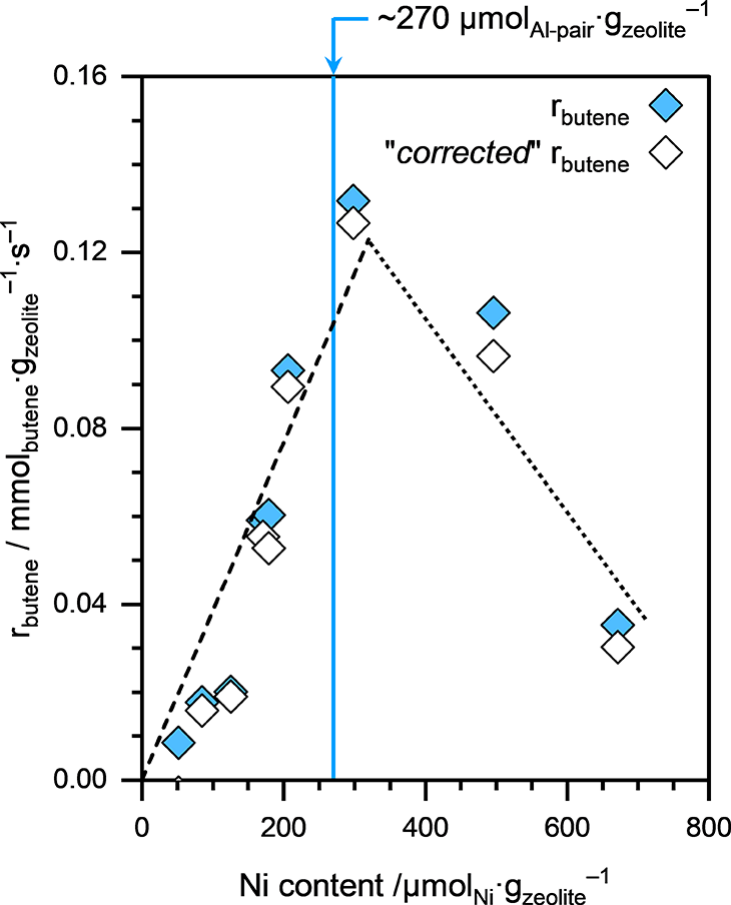
Net and “*corrected*” 1-butene consumption rates (*r*_butene_), normalized to the catalyst mass, on Ni-NaMFI zeolites with different Ni loadings as a function of their Ni content. Reaction conditions: *T* ≈ 433 K, *p*_total_ ≈ 50 bar (15% isobutane and 85% 1-butene), space-velocity: 0.03–0.6 mol_butene_·g_zeolite_^–1^·s^–1^. All reactions were performed under differential (<10%) conversion conditions. The solid blue line represents the Al-pair concentration in the parent HMFI zeolite.

We also performed 1-butene dimerization, under similar supercritical conditions, on the parent HMFI zeolite, and the TOF_butene_ on the BAS in the MFI framework was estimated to be approximately 0.084 mol_butene_·mol_BAS_^–1^·s^–1^. Since all synthesized Ni-NaMFI samples contained non-negligible concentrations of BAS (ranging between 13 and 134 μmol_BAS_·g_zeolite_^–1^), we subtracted their contribution to the net *r*_butene_ obtained on the Ni-NaMFI zeolite samples. The corrected 1-butene consumption rates (denoted as “*corrected*” *r*_butene_) are also presented in Figure 2 as open symbols. From the slope of “*corrected*” *r*_butene_ versus Ni content plot (dashed line; Figure 2), the TOF_butene_ on the isolated Ni^2+^ sites in the MFI framework was estimated to be 0.36 ± 0.04 mol_butene_·mol_Ni_^–1^·s^–1^.

Once Ni loading surpassed the concentration of Al pairs in the Ni-NaMFI zeolite samples, 1-butene consumption rates (when normalized to the catalyst mass) decreased with the increasing Ni content (see the dotted line; Figure 2). This behavior contrasts with that observed on the Ni-NaCHA zeolite samples and is indicative not only of the formation of inactive Ni species in zeolite samples with higher Ni loadings but also of growth of such species at the expense of potential active sites.

In order to elucidate the nature of active Ni sites in the Ni-NaMFI zeolites, we performed in situ XAS measurements at the Ni K-edge (8333 eV) on a representative Ni-NaMFI(206) zeolite sample (with Ni loading below the Al-pair concentration). Prior to the XAS measurements, the zeolite sample was activated in ∼10 vol % O_2_/He at 723 K for 2 h. The X-ray absorption near-edge structures (XANES) of the as-synthesized and activated Ni-NaMFI(206) zeolite samples are presented in Figure 3. As evidenced by the small pre-edge feature at ∼8334 eV in both as-synthesized and activated samples, labeled as “A” in Figure 3 and attributed to the Ni^2+^ 1s → 3d transitions,^42^ we conclude that Ni exists predominantly in the Ni­(II) oxidation state in these Ni-NaMFI zeolites.

**Figure 3:**
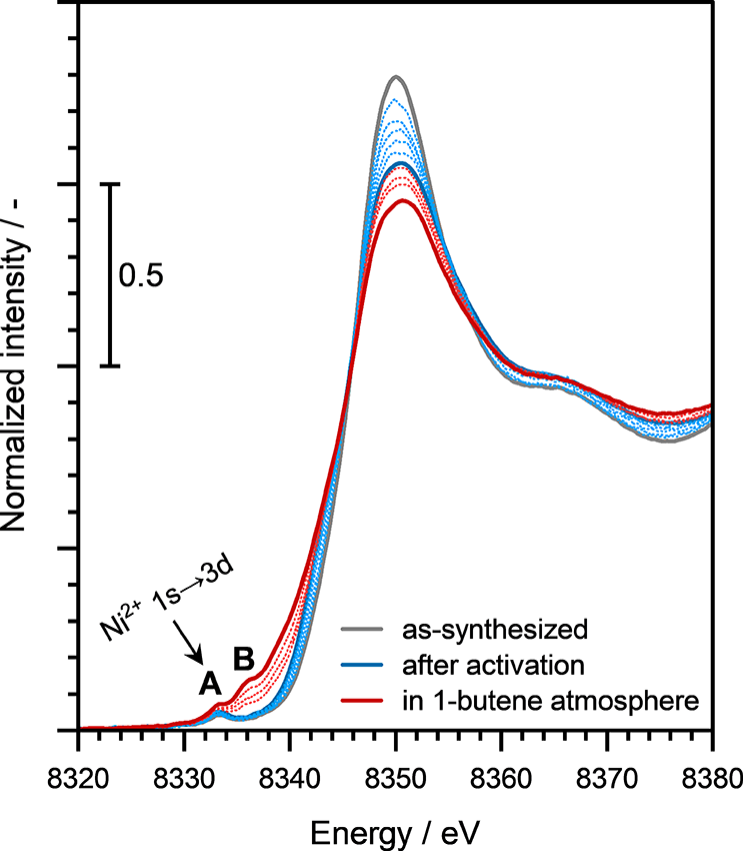
Ni K-edge XANES of the as-synthesized Ni-NaMFI(206) zeolite sample and also after its activation and after exposure to 1-butene. The XANES of the activated sample (solid blue line) was measured in situ after treatment in ∼10 vol % O_2_/He at 723 K for 2 h. The dotted blue lines are spectra measured during the activation procedure. The XANES represented with the solid red line was measured after exposing the catalyst to 1-butene at ∼433 K and ambient pressure for at least 1 h. The dotted red lines are spectra measured during the 1-butene exposure procedure.

The extended X-ray absorption fine structure (EXAFS) of the representative Ni-NaMFI(206) zeolite sample (after activation) is presented in Figure 4. The corresponding EXAFS fitting parameters are summarized in Table 2. The fitted Ni–O and Ni–Al coordination numbers (CN) and interatomic distances (*d*) are indicative of Ni^2+^ cations ion-exchanged at the Al-pair sites in the MFI framework. Additionally, the absence of significant Ni–Ni scattering in the EXAFS results excludes the formation of bulk NiO nanoparticles in this sample. Overall, the XAS results support that, at Ni loadings below the Al-pair exchange limit, the active sites in the Ni-NaMFI zeolites are isolated Ni^2+^ cations, ion-exchanged at the Al-pair sites.

**Figure 4:**
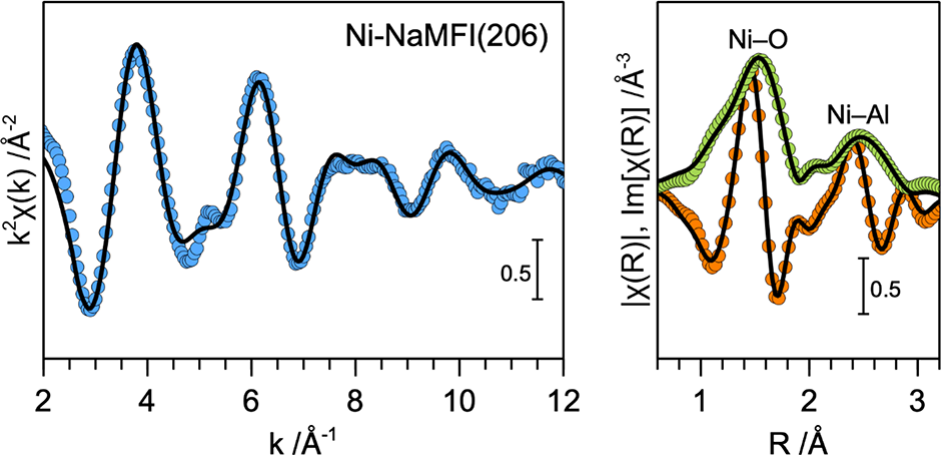
Ni K-edge *k* ^2^-weighted EXAFS (left panel) and FT-EXAFS (right panel) of the activated Ni-NaMFI(206) zeolite sample. Experimental data are shown as closed symbols, and the corresponding fits are shown as solid black lines. The spectra were measured in situ after activation of the catalyst sample in ∼10 vol % O_2_/He at 723 K for 2 h.

**Table 2:** EXAFS Fitting Parameters: CN, Interatomic Distances (*d*), and Debye–Waller Factors (σ^2^) for the Activated Ni-NaMFI(206) Zeolite Sample

path	CN	*d*/Å	σ^2^/Å^2^
Ni–O	4.2 ± 0.6	2.02 ± 0.01	0.006 ± 0.001
Ni–Al	1.6 ± 1.3	2.85 ± 0.06	0.012^a^
Ni–Si	5.8 ± 4.0	3.20 ± 0.03	0.013 ± 0.007

a This parameter was kept fixed during the fitting.

We further investigated the nature of active sites in different Ni-NaMFI zeolite samples using IR spectroscopy of adsorbed CO. The IR spectra of Ni-NaMFI zeolite samples at different CO partial pressures (*p*_CO_), ranging between 0.0001 and 1 mbar, are presented in Supporting Information Figure S5. In the IR spectra, the vibration band at ∼2212 cm^–1^ is attributed to CO adsorbed on the isolated Ni^2+^ cations located at the ion-exchange sites in the MFI zeolite (denoted as Ni^2+^–CO).^28,43,44^ A shoulder to this band, at ∼2204 cm^–1^, is attributed to the formation of dicarbonyl species, also adsorbed on the isolated Ni^2+^ cations (denoted as Ni^2+^–(CO)_2_).^43^ Refer to the Supporting Information Section S3 for detailed peak assignment and analysis of the IR spectra at different *p*_CO_.

Figure 5a shows the intensity of the IR band at ∼2212 cm^–1^, measured at *p*_CO_ ≈ 0.01 mbar for different Ni-NaMFI zeolite samples. This specific *p*_CO_ value was chosen to eliminate any contributions from CO adsorbed on other species or from the partial reduction of Ni^2+^ to Ni^1+^/Ni^0^ (see Supporting Information Figure S5). However, the contribution from the shoulder corresponding to the Ni^2+^–(CO)_2_ species (at ∼2204 cm^–1^) remained present, even at these low CO partial pressures. Therefore, to accurately isolate the intensity of the band associated with CO adsorbed on Ni^2+^ species, the spectra were deconvoluted to exclude the contribution from the Ni^2+^–(CO)_2_ shoulder (for more details, see Supporting Information Figure S6 and Table S6).

**Figure 5:**
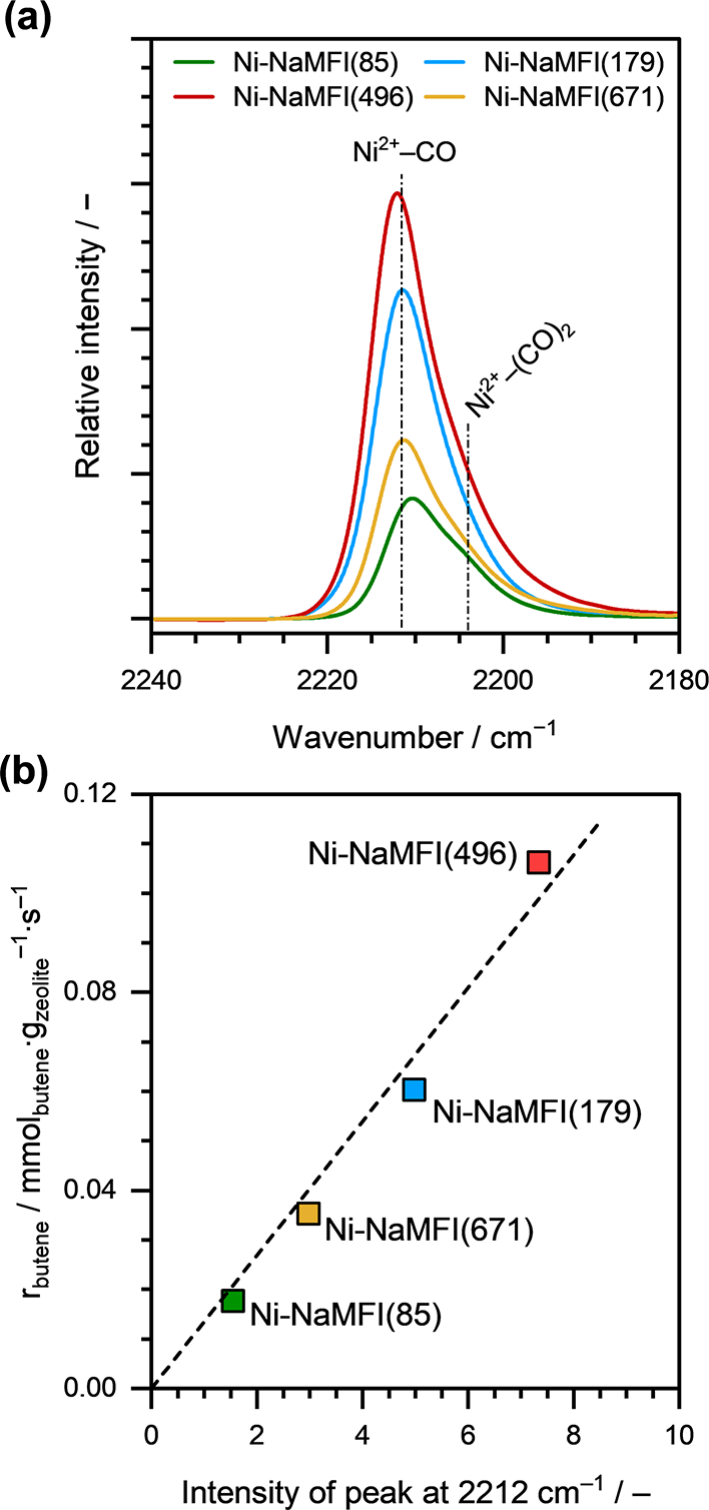
(a) IR spectra of adsorbed CO at *p*_CO_ ≈ 0.01 mbar and liquid N_2_ temperature on Ni-NaMFI zeolite samples with different Ni loadings (ranging between 85 and 671 μmol_Ni_ g_zeolite_^–1^). (b) Rate of 1-butene consumption (*r*_butene_), normalized to the catalyst mass, as a function of the intensity of the Ni^2+^–CO IR band at ∼2212 cm^–1^. Reaction conditions: *T* ≈ 433 K, *p*_total_ ≈ 50 bar (15% isobutane and 85% 1-butene).

Figure 5b shows the 1-butene consumption rates (*r*_butene_), normalized to the catalysts mass, as a function of the intensity of the Ni^2+^–CO vibration band at ∼2212 cm^–1^, for different Ni-NaMFI zeolite samples with Ni loadings ranging between 85 and 671 μmol_Ni_ g_zeolite_^–1^. Notably, these samples include (i) those with Ni loading < Al-pair concentration (270 μmol_Ni_ g_zeolite_^–1^) and (ii) those with Ni loading > Al-pair concentration. The clear linear correlation (dashed line; Figure 5b) confirms that Ni^2+^ cations, exchanged at the Al-pair sites, are indeed the active sites for 1-butene dimerization in Ni-exchanged medium-pore MFI zeolites.

### 1-Butene Dimerization on Ni-NaFAU Zeolites

Now, let us look at 1-butene dimerization on Ni-exchanged large-pore FAU zeolites under supercritical reaction conditions (*T* ≈ 433 K and *p*_butene_ ≈ 42.5 bar). Table 1 presents the total Al content and Si/Al ratio (estimated from AAS) and the concentration of Al pairs (estimated from Co^2+^ ion exchange) in the parent HFAU zeolite. The Ni and Na content, estimated from AAS, as well as the BAS and LAS concentrations, determined from IR spectroscopy of adsorbed pyridine, in the synthesized Ni-NaFAU zeolite samples are summarized in Supporting Information Table S7.

Supporting Information Figure S9a shows *r*_butene_, normalized to the catalyst mass, on Ni-NaFAU zeolite samples as a function of their Ni content under differential (<10%) conversion conditions. In contrast to the Ni-exchanged CHA and MFI zeolites, *r*_butene_ on Ni-NaFAU zeolite samples did not increase linearly with Ni loading but remained almost constant (at ∼4 mol_butene_·g_zeolite_^–1^·h^–1^) in the Ni-loading range of 50–600 μmol_Ni_·g_zeolite_^–1^. Figure 6 presents TOF_butene_, normalized to the total Ni content, for Ni-NaFAU zeolite samples as a function of the total Ni content. Obviously, the constant 1-butene dimerization activity per gram of catalyst implies that the TOF_butene_ decreased with increasing Ni content in the Ni-NaFAU zeolite samples (dashed line; Figure 6).

**Figure 6:**
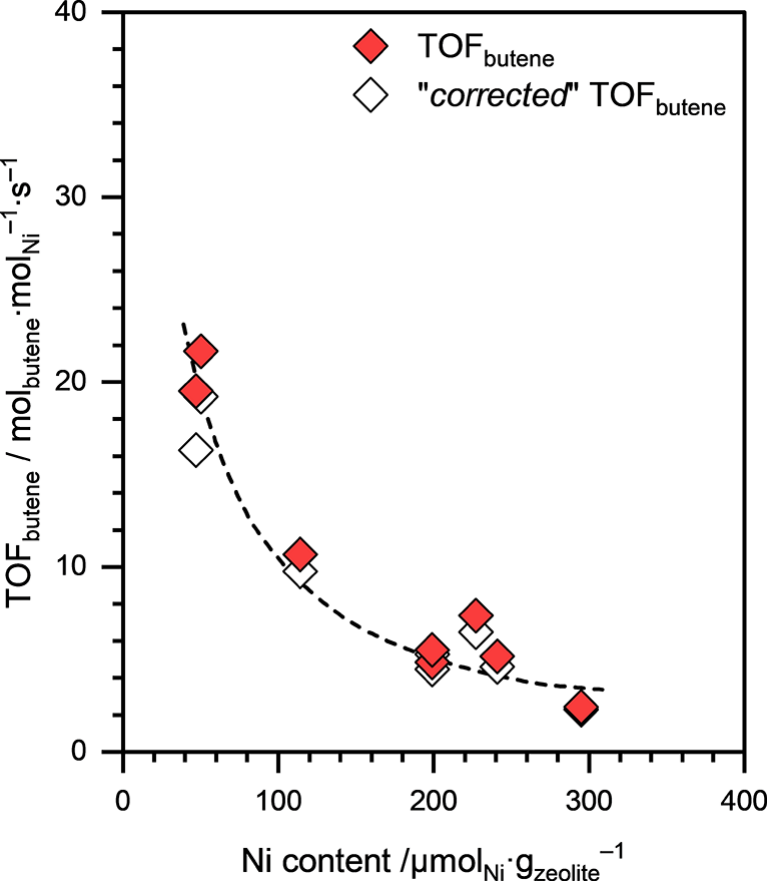
TOF_butene_ (filled symbols) and “*corrected*” TOF_butene_ (open symbols) on Ni-NaFAU zeolite samples with different Ni loadings as a function of their Ni content. Reaction conditions: *T* ≈ 433 K, *p*_total_ ≈ 50 bar (15% isobutane and 85% 1-butene). All reactions were performed under differential (<10%) conversion conditions.

Next, we investigated the coordination structure of Ni species formed in these ion-exchanged Ni-NaFAU zeolite samples using in situ Ni K-edge XAS. The XANES of these samples (discussed later) indicated that Ni exists predominantly in the Ni­(II) oxidation state. The EXAFS of three zeolite samples with different Ni loadings, viz., Ni-NaFAU(50), Ni-NaFAU(200), and Ni-NaFAU(607), are presented in Figure 7, and the corresponding EXAFS fitting parameters are summarized in Table 3. Notably, no Ni–Ni scattering was observed in the EXAFS of Ni-NaFAU(50), the sample with the lowest Ni loading. The absence of Ni–Ni scattering excludes the presence of bulk NiO species in this sample. The CN_Ni–Al_ ≈ 2 suggests that in this zeolite sample, Ni predominantly exists as isolated Ni^2+^ species exchanged at the Al-pair sites.

**Figure 7:**
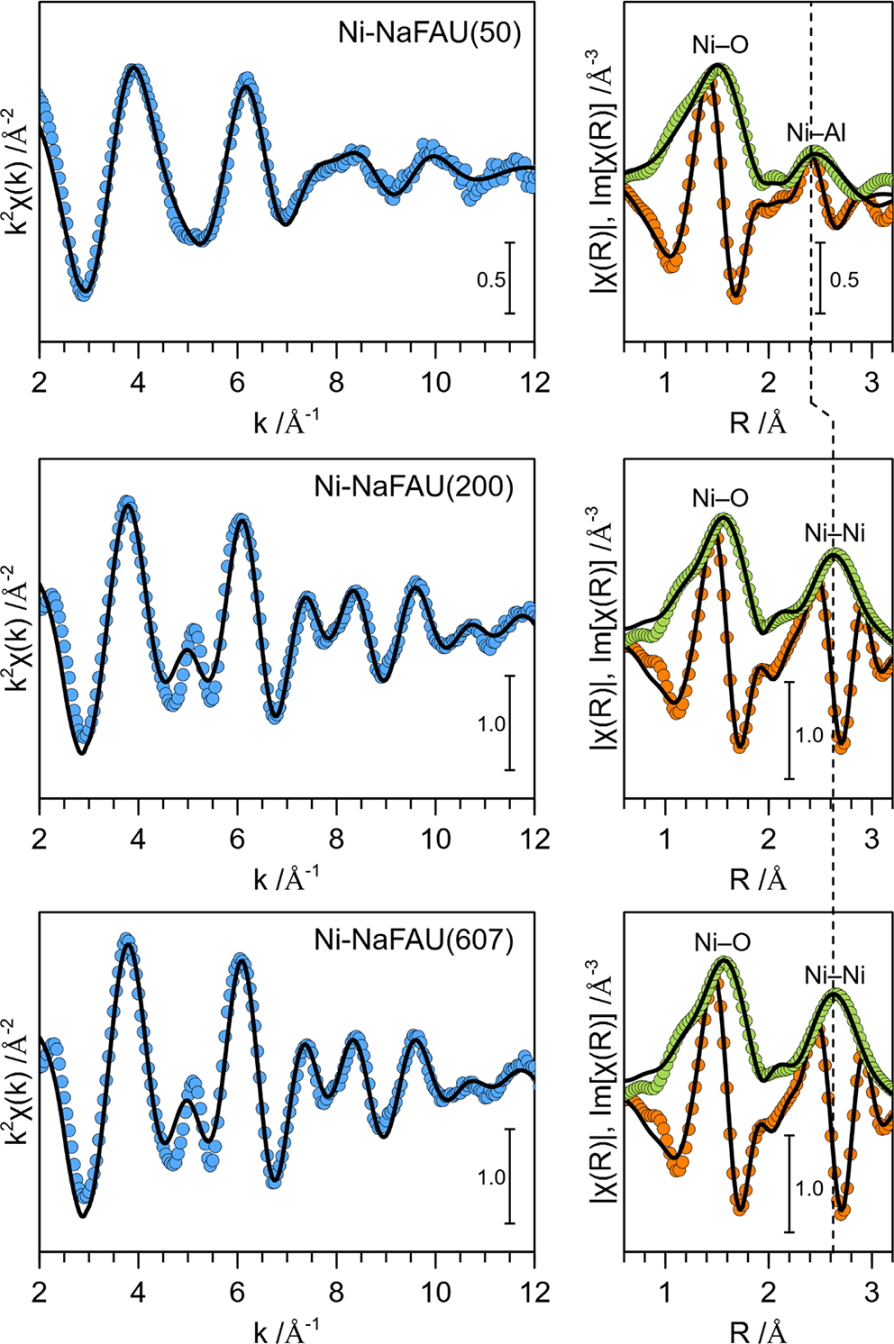
Ni K-edge *k* ^2^-weighted EXAFS (left panel) and FT-EXAFS (right panel) of activated Ni-NaFAU(50), Ni-NaFAU(200), and Ni-NaFAU(607) zeolite samples. Experimental data are shown as closed symbols, and the corresponding fits are shown as solid black lines. All spectra were measured in situ after activation of the catalysts in ∼10 vol % O_2_/He at 723 K for 2 h.

**Table 3:** EXAFS Fitting Parameters: CN, Interatomic Distances (*d*), and Debye–Waller Factors (σ^2^) for Ni-NaFAU Zeolite Samples with Different Ni Loadings

catalyst	path	CN	*d*/Å	σ^2^/Å^2^
Ni-NaFAU(50)	Ni–O	∼3.8	∼2.00	∼0.009
	Ni–Al	∼1.8	∼2.84	0.012^a^
	Ni–Si	∼3.4	∼3.20	∼0.018
Ni-NaFAU(200)	Ni–O	∼4.8	∼2.05	∼0.007
	Ni–Ni	∼5.7	∼3.06	∼0.011
	Ni–Al	∼1.9	∼2.70	∼0.014
Ni-NaFAU(600)	Ni–O	∼5.0	∼2.04	∼0.007
	Ni–Ni	∼8.3	∼3.07	∼0.013
	Ni–Al	∼2.1	∼2.67	0.017^a^

a This parameter was kept fixed during the fitting.

In contrast to Ni-NaFAU(50), samples with a higher Ni content, i.e., Ni-NaFAU(200) and Ni-NaFAU(607), show a dominant Ni–Ni scattering contribution at *d* ≈ 3.06 Å. For reference, the Ni–Ni interatomic distance in bulk nickel oxide is 2.95–2.98 Å. Moreover, the Ni–O CNs and interatomic distances in Ni-NaFAU(200) and Ni-NaFAU(607) were similar to those for bulk nickel oxide (CN_Ni–O_ = 6 at *d*_Ni–O_ = 2.08 Å). The structural parameters deduced from EXAFS analysis suggest the formation of slightly distorted bulk NiO nanoparticles in these zeolite samples. It is also worth mentioning that the theoretical CN_Ni–Ni_ in bulk NiO is equal to 12. Therefore, the slightly lower CN_Ni–Ni_ values for these samples (∼5.7 for Ni-NaFAU(200) and ∼8.3 for Ni-NaFAU(607)) suggest that (i) the bulk NiO nanoclusters formed in the Ni-NaFAU zeolites are small and (ii) their size increases with increasing Ni loading.

Based on the systematic decrease in TOF_butene_ with increasing Ni content (see Figure 6), combined with the structural insights from EXAFS analysis, we postulate that isolated Ni^2+^ cations, ion-exchanged at the Al-pair sites, are the active sites for 1-butene dimerization in the Ni-NaFAU zeolite sample with the lowest Ni loading. In samples with Ni loadings >50 μmol_Ni_·g_zeolite_^–1^, Ni is predominantly deposited as inactive bulk NiO nanoclusters.

We also performed 1-butene dimerization on the parent HFAU zeolite under supercritical reaction conditions, and the TOF_butene_ on the BAS in FAU zeolite was estimated to be ∼1.7 mol_butene_·mol_BAS_·s^–1^, significantly higher than the TOF_butene_ on the BAS in CHA or MFI zeolites. Since the synthesized Ni-NaFAU zeolites contained a non-negligible concentration of BAS (ranging between 24 and 130 μmol_BAS_·g_zeolite_^–1^), we subtracted their contribution to the net *r*_butene_ exhibited by the Ni-NaFAU zeolite samples. The “corrected” *r*_butene_ values are presented in Figure 6 as open symbols. The lower bound for the “corrected” TOF_butene_ on the active Ni sites in the FAU zeolite was estimated to be ∼19.2 mol_butene_·mol_Ni_·s^–1^. This value is almost two-orders-of-magnitude higher than the TOF_butene_ on the Ni sites in the Ni-exchanged CHA and MFI zeolite samples.

### 1-Butene Dimerization on Ni-HFAU Zeolites

To understand the effect of Na^+^ presence on the Ni^2+^ ion exchange capacity of the FAU zeolites, we exchanged the H-form of the parent FAU zeolite directly with Ni^2+^ cations, i.e., without prior Na^+^ exchange. Supporting Information Figure S9b shows *r*_butene_, normalized to the catalyst mass, on Ni-NaFAU zeolite samples as a function of their Ni content under differential (<10%) conversion conditions. Figure 8 shows the TOF_butene_, normalized to the total Ni content, for Ni-HFAU zeolite samples with different Ni loadings as a function of their Ni content. Similar to the Ni-NaFAU series, the TOF_butene_ on Ni-HFAU zeolites also decreased systematically with increasing Ni loading (see dashed line; Figure 8). We also subtracted the contribution of BAS present in Ni-HFAU zeolite samples to the 1-butene dimerization activity, and the “corrected” TOF_butene_ for the Ni-HFAU zeolites, as a function of their Ni content, are presented in Figure 8. The “corrected” TOF_butene_ for Ni-HFAU zeolite samples increased initially, reached a maximum of ∼12 μmol_butene_·mol_Ni_^–1^·s^–1^ at a Ni loading of ∼55 μmol_Ni_·g_cat_^–1^, and then decreased as the Ni loading increased further (see solid line; Supporting Information Figure S8).

**Figure 8:**
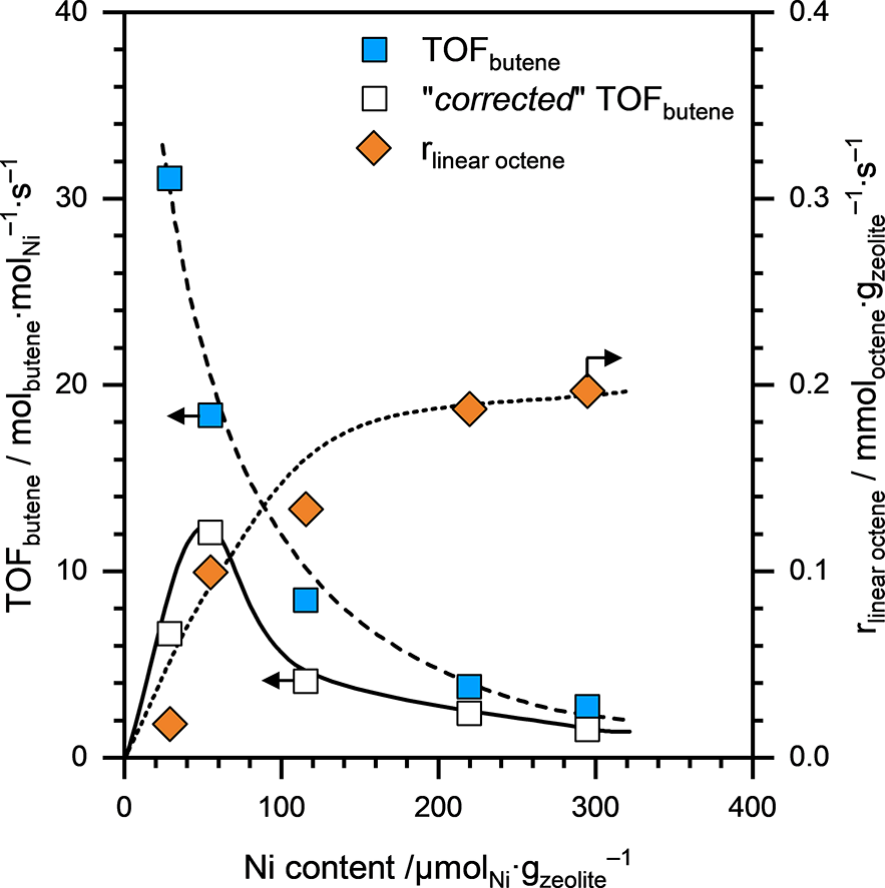
TOF_butene_ (blue squares) and “corrected” TOF_butene_ (open squares) and linear octene formation rates (*r*_linearoctene_; orange diamonds) on Ni-HFAU zeolite samples with different Ni loadings as a function of their Ni content. Reaction conditions: *T* ≈ 433 K, *p*_total_ ≈ 50 bar (15% isobutane and 85% 1-butene). All reactions were performed under differential (<10%) conversion conditions. The lines are visual guides.

Next, to compare the nature of ion-exchanged Ni^2+^ cations in the Ni-NaFAU and Ni-HFAU zeolites, we performed in situ XAS on these samples at the Ni K-edge. The XANES and the EXAFS of the as-synthesized Ni-NaFAU and Ni-HFAU zeolite samples are presented in Supporting Information Figures S7 and S8, respectively. The XANES of Ni-NaFAU and Ni-HFAU zeolite samples with different Ni loadings after activation is presented in Figure 9. As evidenced by the presence of Ni^2+^ 1s → 3d transitions at ∼8334 eV (labeled as “A”; Figure 9), we conclude that Ni predominantly exists in the Ni­(II) oxidation state in these catalysts. Additionally, the XANES of the activated Ni-NaFAU zeolite samples with higher Ni loadings exhibited multiple-scattering features at ∼8353 eV and ∼8370 eV, which can be attributed to the formation of NiO clusters (labeled as “D”; Figure 9a).^45^ Notably, the Ni-NaFAU(50) zeolite sample did not exhibit these features, further confirming (in agreement with the EXAFS analysis) that Ni exists predominantly as isolated Ni^2+^ cations in this sample. The XANES and the EXAFS of the as-synthesized zeolite samples are also in agreement with this conclusion (see Supporting Information Figures S7 and S8).

**Figure 9:**
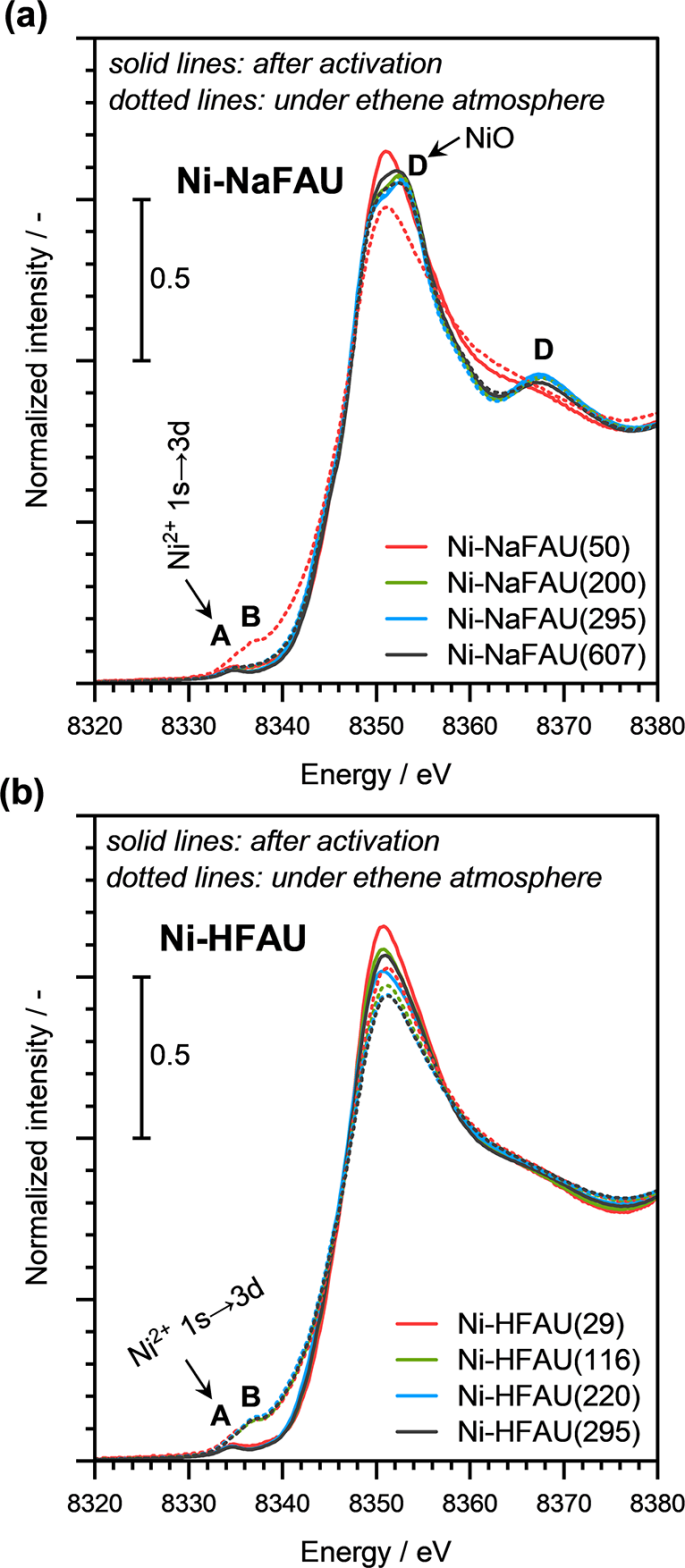
Ni K-edge XANES of (a) Ni-NaFAU (top panel) and (b) Ni-HFAU (bottom panel) zeolite samples with different Ni loadings. Spectra measured after activation (in 10 vol % O_2_/He at 723 K for 2 h) are shown as solid lines. Spectra measured under an ethene atmosphere (ambient temperature and pressure) are shown as dashed lines. The labeled features correspond to, “A”: Ni^2+^ 1s → 3d, “B”: partially reduced Ni^2+^ species, “D”: bulk NiO.

Interestingly, in contrast to the Ni-NaFAU samples, the XANES of Ni-HFAU zeolite samples (see Figure 9b) did not exhibit the characteristic multiple scattering features at ∼8353 eV and ∼8370 eV. These features were absent even in the Ni-HFAU zeolite sample with the highest Ni loading that we investigated (with ∼295 μmol_Ni_·g_cat_^–1^). Moreover, the XANES and the EXAFS of the as-synthesized Ni-HFAU samples also excluded the formation of bulk NiO clusters in these materials (see Supporting Information Figures S7 and S8). Overall, the XAS analysis clearly suggests that Ni is primarily present as an isolated Ni species at the ion exchange sites in the Ni-HFAU zeolites. Moreover, this is the case even for the sample with the highest Ni content. These findings also imply that in comparison to the Ni-NaFAU zeolites, the tendency to form bulk NiO nanoparticles is relatively low for the Ni-HFAU zeolites.

Next, we investigated the adsorption of alkenes on the Ni sites present in these Ni-exchanged FAU zeolites using in situ XAS. For this, we exposed the activated Ni-NaFAU and Ni-HFAU zeolite samples to ethene at ambient pressure and temperature and measured the adsorption with in situ XAS. It must be noted here that we used ethene instead of 1-butene due to experimental consideration at the synchrotron radiation facility. However, we expect the electronic changes incurred on Ni^2+^ sites due to π- or σ-interactions to be similar for ethene and 1-butene.

The in situ measured XANES of Ni-NaFAU zeolite samples in the presence of ethene are presented in Figure 9a as dotted lines. Interestingly, for Ni-NaFAU(50), we observed a partial reduction of Ni^2+^ upon exposure to ethene, as evidenced by the additional pre-edge feature at ∼8337 eV (labeled as “B”, Figure 9a). The partial reduction of Ni^2+^ in the presence of an alkene, in this case, ethene, is likely due to the electron density transfer from the π- or σ-bonded alkene molecules. However, this reduction was evident only for the Ni-NaFAU sample with the lowest Ni loading, i.e., Ni-NaFAU(50). In the case of Ni-NaFAU zeolite samples with Ni loading >50 μmol_Ni_·g_zeolite_^–1^, we did not observe the pre-edge feature at ∼8337 eV upon exposure to ethene. This is hypothesized to be due to the fact that the majority of the Ni^2+^ sites, existing as inactive Ni^2+^ cations in the bulk NiO nanoparticles in these materials, are not influenced by π- or σ-bond interactions with an alkene.

The Ni K-edge XANES of Ni-HFAU zeolite samples, measured after exposure to ethene, is presented as dotted lines in Figure 9b. Remarkably, for all Ni-HFAU zeolite samples, even the ones with the highest Ni loading, we observed partial reduction of Ni^2+^ in the presence of ethene, as evidenced by the pre-edge feature at ∼8337 eV (labeled as “B”; Figure 9a). We also recall here that the Ni K-edge XANES and EXAFS of the as-synthesized Ni-HFAU zeolite samples also did not indicate the presence of any bulk NiO clusters in these materials (see Supporting Information Figures S7 and S8). Therefore, XAS analysis further suggests that Ni is homogeneously dispersed in the Ni-HFAU zeolite as Ni^2+^ cations at accessible positions within the zeolite even at higher Ni loadings.

Now let us discuss the activity of Ni^2+^ sites in the Ni-HFAU zeolites for the 1-butene dimerization reaction. We have noted above that the 1-butene dimerization activity on Ni-HFAU zeolite samples includes significant contributions from the BAS present in the zeolite. However, it is also worth noting here that the dimerization of 1-butene on active Ni sites results in the formation of linear octenes.^8^ On the other hand, starting from 1-butene as the reactant, the formation of linear octenes is not favored by the acid-catalyzed pathway.^8^ In other words, the dimerization of 1-butene on BAS does not form linear octenes. Therefore, we used linear octene formation to assess the contribution of Ni^2+^ sites to the dimerization activity of the synthesized Ni-HFAU zeolite samples.

Figure 8 also presents the rate of formation of linear octenes ( $r_{linear\;octene}$ ), normalized to the catalyst mass, for different Ni-HFAU zeolite samples with varying Ni loading. We can clearly see that $r_{linear\;octene}$ increased systematically with increasing Ni content, indicating the formation of active Ni^2+^ sites that are accessible to 1-butene (dotted line; Figure 8). Notably, $r_{linear\;octene}$ almost plateaued at a Ni loading of ∼200 μmol_Ni_·g_zeolite_^–1^, suggesting that this Ni loading results in the maximum concentration of isolated Ni^2+^ cations that are accessible to 1-butene. Additionally, the decreasing TOF_butene_ as a function of Ni content (dashed line; Figure 8) suggests that not all Ni sites are accessible to 1-butene. Based on these observations, we conclude that in the Ni-HFAU zeolite series, Ni^2+^ cations are exchanged at several potential locations within the FAU framework, with only some locations resulting in Ni^2+^ sites that are accessible to 1-butene. These locations are fully occupied at Ni loadings above ∼200 μmol_Ni_·g_zeolite_^–1^ in HFAU while in NaFAU, the maximum site concentration is achieved at a loading of ∼50 μmol_Ni_·g_zeolite_^–1^ or lower.

In relevant literature, S_I_ and S_II_ sites of the FAU framework (see Supporting Information Figure S10) have been described as the preferred ion-exchange locations for divalent cations like Ni^2+^, especially at low loadings.^46,47^ The S_II_ sites are located in the plane of the six-membered rings shared between the sodalite cage and the faujasite supercage. These S_II_ sites are accessible from the faujasite supercage, which allows enough space for the butene dimerization’s bulky intermediates. Conversely, the S_I_ sites, located in the center of the hexagonal prism, are not accessible directly from the faujasite supercage. The S_I_ and S_II_ sites have also been suggested as the preferred exchange sites for monovalent cations like Na^+^.^48,49^

We propose that in the Ni-NaFAU zeolites, the presence of Na^+^ cations hinders the ion exchange of Ni^2+^ at S_I_ sites and, therefore, Ni is preferentially ion-exchanged at the S_II_ sites at low Ni loadings. Additionally, since the S_I_ sites are occupied by Na^+^ cations, formation of bulk nickel oxide clusters occurs, even in the zeolite samples with relatively low Ni loading (>50 μmol_Ni_·g_zeolite_^–1^). A certain fraction of Ni^2+^ cations, exchanged in the S_II_ sites, converts 1-butene with a very high TOF_butene_ (at least 19.2 mol_butene_·mol_Ni_·s^–1^). Conversely, in the Ni-HFAU zeolites, because there is no competition with Na^+^, a relatively large concentration of Ni is preferentially ion-exchanged, as isolated Ni^2+^ cations, at the preferred S_I_ sites. However, these Ni^2+^ cations present at S_I_ sites are inaccessible to 1-butene and, therefore, do not contribute to the overall activity of the Ni-HFAU series. However, similar to the Ni-NaFAU series, a certain fraction of Ni in the Ni-HFAU zeolites also ion exchanges at the accessible S_II_ sites (reaching a maximum at ∼200 μmol_Ni_·g_zeolite_^–1^) and causes the overall dimerization of 1-butene on these catalysts.

### Effect of Local Environment around Ni^2+^ Cations on 1-Butene Dimerization Selectivity

The selectivity trends of 1-butene dimerization toward C_8_= isomers reveal mechanistic details as well as shape selectivity of the different zeolite frameworks. The dimerization of 1-butene on Ni^2+^ sites primarily leads to a mixture of only linear octenes and methylheptenes as the primary products.^8^ On the other hand, Brønsted acid-catalyzed alkene double-bond isomerization and dimerization favors branched products such as dimethylhexenes.^8^ Additionally, dimerization of 1-butene on Ni sites via the Cossee–Arlman-type mechanism does not form dimethylhexene.^8^ However, it has been suggested that dimerization of 2-butene on the Ni sites can form dimethylhexene as a product.^8^ Figure 10 presents the selectivity toward C_8_= isomers (categorized as linear octenes, methylheptenes, and dimethylhexenes) and C_12_= as a function of 1-butene conversion on different Ni-exchanged CHA, MFI, and FAU zeolite samples.

**Figure 10:**
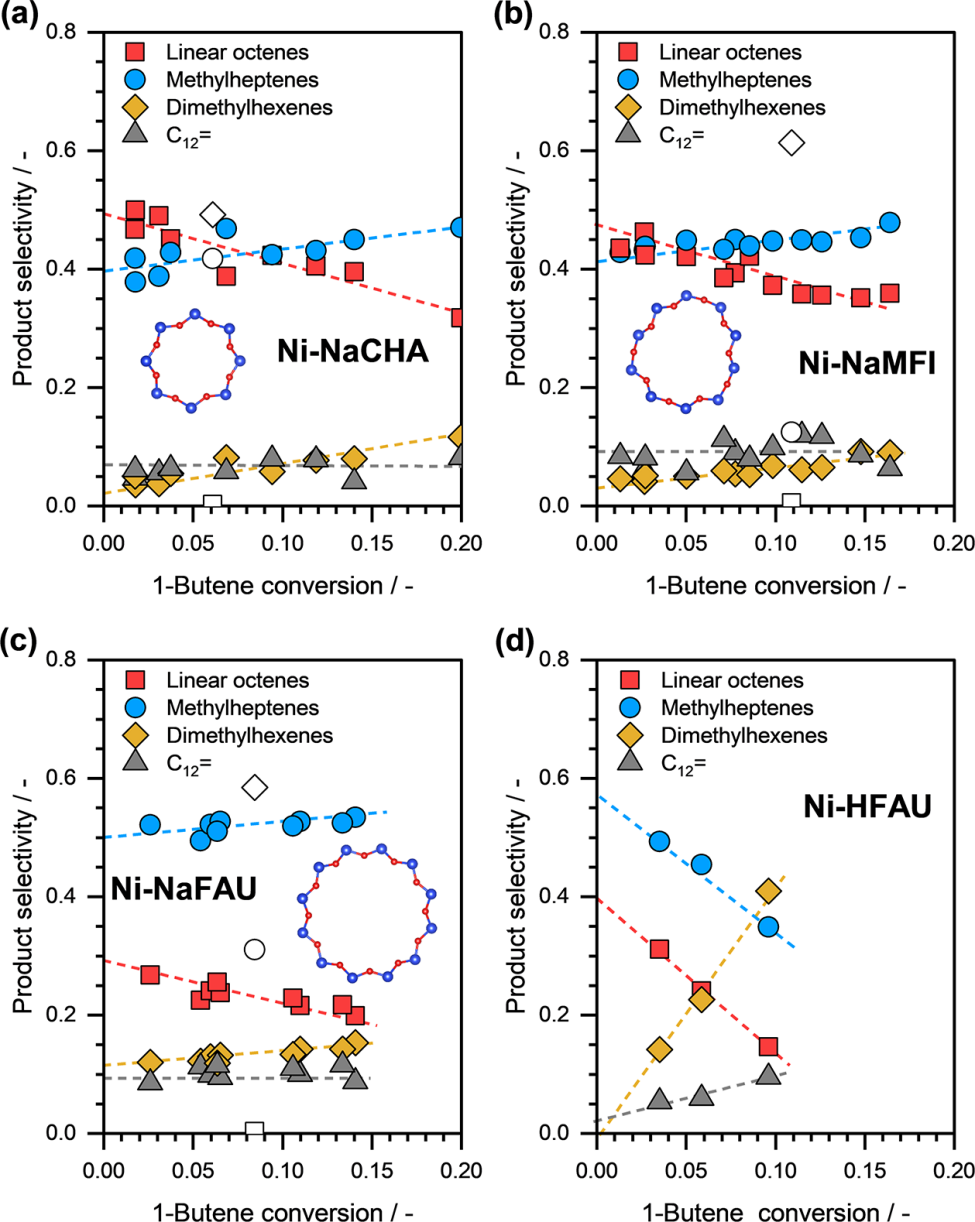
1-Butene dimerization selectivity toward linear octenes (red squares), methylheptenes (blue circles), dimethylhexenes (yellow diamonds), and C_12_= isomers (gray triangles) as a function of 1-butene conversion on representative (a) Ni-NaCHA, (b) Ni-NaMFI, (c) Ni-NaFAU, and (d) Ni-HFAU zeolite samples. 1-Butene dimerization selectivity on parent HCHA, HMFI, and HFAU zeolite is presented as open symbols. Reaction conditions: *T* ≈ 433 K, *p*_total_ ≈ 50 bar (15% isobutane and 85% 1-butene). Inset: pore openings of CHA, MFI, and FAU zeolite frameworks. Si/Al: blue, O: red.

For Ni-NaCHA zeolite samples, at low 1-butene conversions, the selectivity toward linear octenes reached almost 50% (see Figure 10a). The obtained linear octenes selectivity on Ni-NaCHA was comparable to the performance of the Ni–Ca-LTA.^26^ The linear octene selectivity on Ni-NaMFI zeolites was also relatively high, almost 47% at low 1-butene conversions (see Figure 10b). We must note here in passing that the selectivity toward linear octenes was lower for Ni-NaCHA and Ni-NaMFI zeolite samples with lower Ni content, which can be attributed to the enhanced isomerization of 1-butene to 2-butene (on Ni sites or residual BAS) at lower space velocities required for comparable conversion. In contrast to Ni-NaCHA and Ni-NaMFI, the Ni-NaFAU and Ni-HFAU zeolite samples exhibited relatively low linear octene selectivity, almost 30% at low 1-butene conversions (see Figure 10c,d). Notably, the selectivity toward dimethylhexenes was negligible, at low 1-butene conversion, on these Ni-exchanged zeolite samples under the investigated reaction conditions.

Based on these results, we can see that the selectivity toward linear octenes decreased in the following order: Ni-NaCHA > Ni-NaMFI ≫ Ni-NaFAU. This trend is attributed to the different pore-opening sizes in the corresponding zeolite frameworks, which increase as follows: CHA (3.8 Å × 3.8 Å) < MFI (5.5 Å × 5.1 Å) ≪ FAU (7.4 Å × 7.4 Å). Overall, our findings clearly indicate that smaller pore openings in zeolites enhance the selectivity toward linear isomers by limiting the diffusion of branched products. Furthermore, the non-negligible selectivity toward dimethylhexenes (at least 4–5%), even at low 1-butene conversions, in all Ni-exchanged zeolites can be attributed to partial isomerization of 1-butene to 2-butene on the Ni sites, prior to the dimerization step.

Figure 10a–c also presents the 1-butene dimerization selectivity obtained on the parent HCHA, HMFI, and HFAU zeolite samples, as open symbols. As expected, the parent H-form of the zeolites, which contain a significant concentration of BAS, exhibited high selectivity toward the branched isomers, viz., dimethylhexenes and methylheptenes, and negligible selectivity toward linear octenes. These results, therefore, further highlight the ability of isolated Ni^2+^ cations to dimerize 1-butene with high selectivity toward the linear C_8_= products.

In all the zeolite samples that we investigated, the linear octenes selectivity decreased systematically with increasing 1-butene conversion, while the selectivity toward methylheptenes and dimethylhexenes increased. The lower linear octenes selectivities at higher conversions is likely due to the progress in butene double bond isomerization (from 1-butene to 2-butene), which is also catalyzed by the Ni sites, or subsequent skeletal isomerization of the products on the residual BAS.^8^ The residual BAS in the Ni-NaCHA zeolites may also contribute to double bond isomerization and the formation of branched isomers. We also note that in the case of Ni-HFAU zeolite sample, the linear octenes selectivity decreased considerably faster with increasing 1-butene conversion (see Figure 10d). We attribute this faster decrease to the presence of a large number of BAS in the Ni-HFAU zeolite sample that contributes to the skeletal isomerization of linear isomers to branched isomers and also to the skeletal and double-bond isomerization of 1-butene.

### Mechanism of 1-Butene Dimerization on Isolated Ni Species

The TOF_butene_ values on the isolated Ni sites in the Ni-exchanged samples with different zeolite frameworks are compared in Table 4. The TOF_butene_ on Ni sites was highest for the FAU framework (∼19.2 mol_butene_·mol_Ni_^–1^·s^–1^), almost two-orders-of-magnitude higher than the TOF_butene_ reported for MFI or CHA zeolites (0.33–0.36 mol_butene_·mol_Ni_^–1^·s^–1^). Based on our characterization (XAS and IR spectroscopy) and kinetic investigations, we have concluded that the active site for 1-butene dimerization is similar in all three zeolites: i.e., isolated Ni^2+^ cations exchanged at the Al-pair locations. Therefore, we postulate that the local environment around the isolated Ni^2+^ cations in these different zeolite frameworks leads to a significant difference in 1-butene dimerization TOF.

**Table 4:** TOF (in mol_butene_·mol_Ni_^–1^·s^–1^), Apparent Activation Energies (*E*_A,app_), Reaction Orders (Ro), Relative Adsorption Enthalpies (ΔΔ*H*_ads_), and Relative Intrinsic Activation Energies (Δ*E*_A,int_) for Ni-Exchanged FAU, MFI, and CHA Zeolites

zeolite	TOF	*E*_A,app_/kJ mol^–1^	ΔΔ*H*_ads_ ^a^/kJ mol^–1^	Δ*E*_A,int_ ^b^/kJ mol^–1^
FAU	∼19.2	–43		
CHA	∼0.33	–32	–10 ± 2	21 ± 2
MFI	∼0.36	–27	–20 ± 4	36 ± 4

a Relative to the Δ*H*_ads_ of 1-butene on FAU.

b Relative to the *E*_A,int_ on FAU.

Figure 11a presents the Arrhenius-type plots for 1-butene dimerization on three representative Ni-NaCHA(174), Ni-NaMFI(179), and Ni-NaFAU(50) zeolite samples. The apparent activation energies (*E*_A,app_) estimated from these Arrhenius-type plots are reported in Table 4. For all three zeolite samples, *r*_butene_ decreased with increasing temperature, thus, resulting in negative *E*_A,app_ values. This is attributed to a strong exothermic adsorption step prior to the overall rate-determining step for the reaction. Figure 11b shows TOF_butene_ as a function of 1-butene partial pressures (*p*_butene_) on Ni-NaCHA(174), Ni-NaMFI(179), and Ni-NaFAU(50) zeolite samples. The reaction orders in *p*_butene_ (ro_butene_) were estimated from these plots and are reported in Table 4. The ro_butene_ values between one and two are expected for olefin dimerization reactions.^8,24,34,50,51^

**Figure 11:**
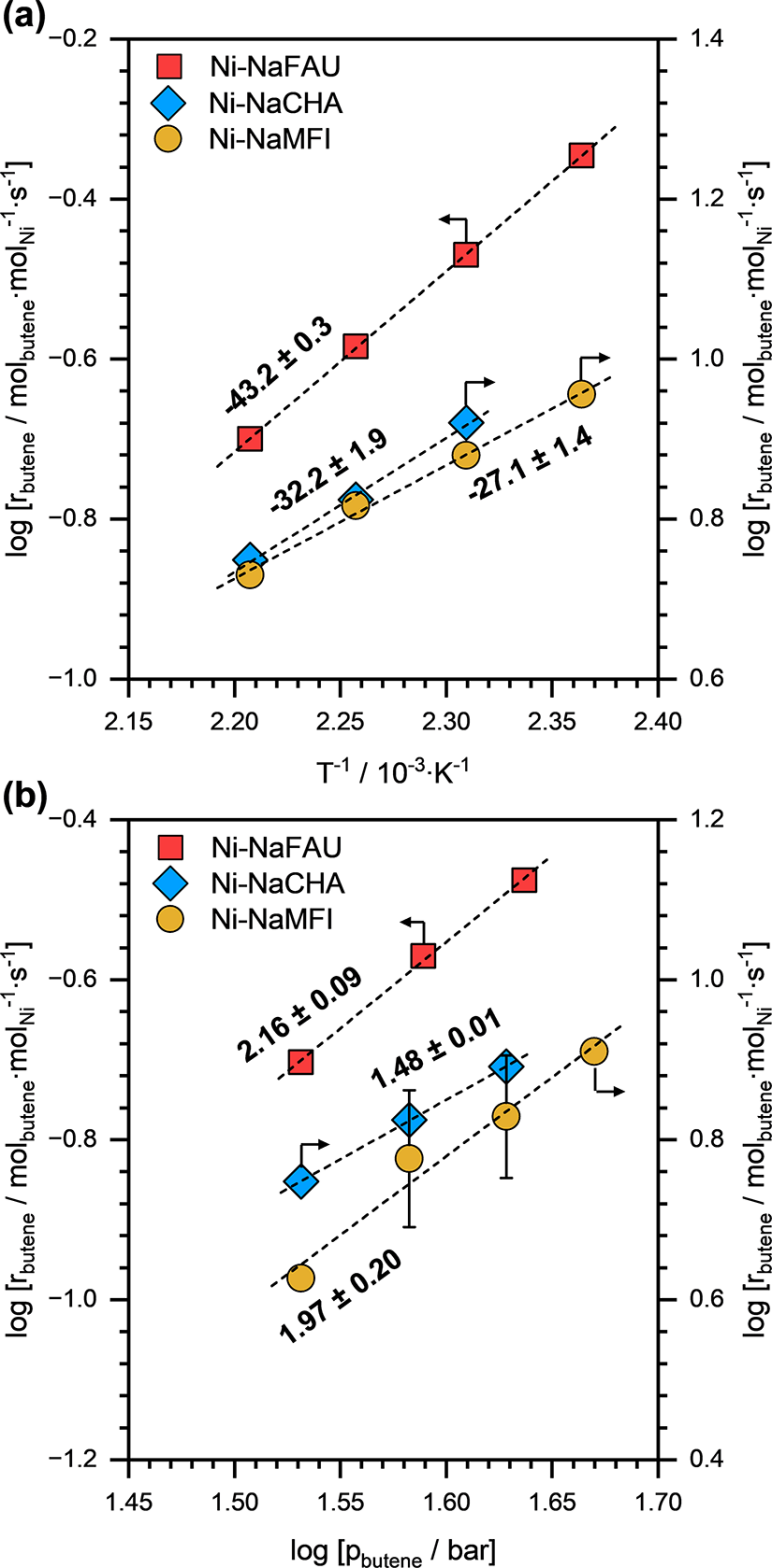
(a) Arrhenius-type plots for 1-butene dimerization on Ni-NaCHA(174), Ni-NaMFI(179), and Ni-NaFAU(50) zeolite samples. The reported numbers are apparent activation energies (*E*_A,app_) in kJ·mol^–1^. (b) *r*_butene_ as a function of *p*_butene_ on Ni-NaCHA(174), Ni-NaMFI(179), and Ni-NaFAU(50) zeolite samples. The reported number are the reaction orders in *p*_butene_. Reaction conditions: *T* ≈ 323–353 K, *p*_total_ ≈ 40–55 bar (15% isobutane and 85% 1-butene). All reactions were performed at differential (<10%) conversion conditions.

Based on the observed kinetic parameters, combined with characterization results, we now address the possible reaction mechanisms of 1-butene dimerization on the isolated Ni-sites in Ni-exchanged zeolites. Several mechanisms have been proposed in literature describing alkene oligomerization on metal-exchanged zeolites.^37^ The proposed mechanisms can be broadly classified as (i) a coordination-insertion mechanism (or Cossee–Arlman-type mechanism) or (ii) a metallacycle mechanism. In the Cossee–Arlman-type mechanism (for butene dimerization on Ni), the dimer formation proceeds via the insertion of an adsorbed butene molecule into the Ni–carbon bond of a Ni-butyl or a Ni-butenyl complex. In the metallacycle mechanism, dimer formation proceeds via the formation of a cyclopentane intermediate with the metal center.

Brogaard and Olsbye,^39^ based on their theoretical calculations, suggested the metallacycle mechanism to be thermodynamically unfavorable on Ni-exchanged zeolites. Additionally, the metallacycle mechanism predominantly results in the formation of dimers; the formation of trimeric species is less likely. In all Ni-exchanged zeolites investigated here, we observed, however, almost 10% selectivity toward C_12_= products (see Figure 10), even at low 1-butene conversions. Therefore, based on the non-negligible selectivity toward trimers, we postulate that the butene dimerization proceeds via a Cossee–Arlman-type mechanism, likely on in situ generated Ni-butyl-type complexes as the active centers.^8,10,37,39,52^ Although the exact mechanism of formation of such Ni-alkyl complexes is not well understood, these complexes likely form upon the interaction of a 1-butene molecule with a Ni^2+^-H-like species.

A schematic of the proposed mechanism, proceeding on in situ formed Ni-butyl complexes (**1a**), is illustrated in Scheme 1. Based on the experimentally obtained negative *E*_a,app_ values and assuming that the C–C bond formation is the kinetically rate-determining step, we postulate that the C–C coupling transition state (**1c**; Scheme 1) is preceded by the adsorption of a 1-butene molecule on the Ni-butyl complex (**1b**; Scheme 1). The reactant 1-butene molecules likely interact with Ni via π-interactions. Such an interaction must potentially increase the electron density around the Ni^2+^ cations, thus resulting in their partial reduction. This reduction was evident in the Ni K-edge XANES of the Ni-NaMFI(206) zeolite sample, measured in situ under a 1-butene atmosphere at 433 K (solid red line; Figure 3), and on the Ni-HFAU and Ni-NaFAU samples, measured under an ethene atmosphere at room temperature (see dotted lines; Figure 8).

**Scheme 1:**
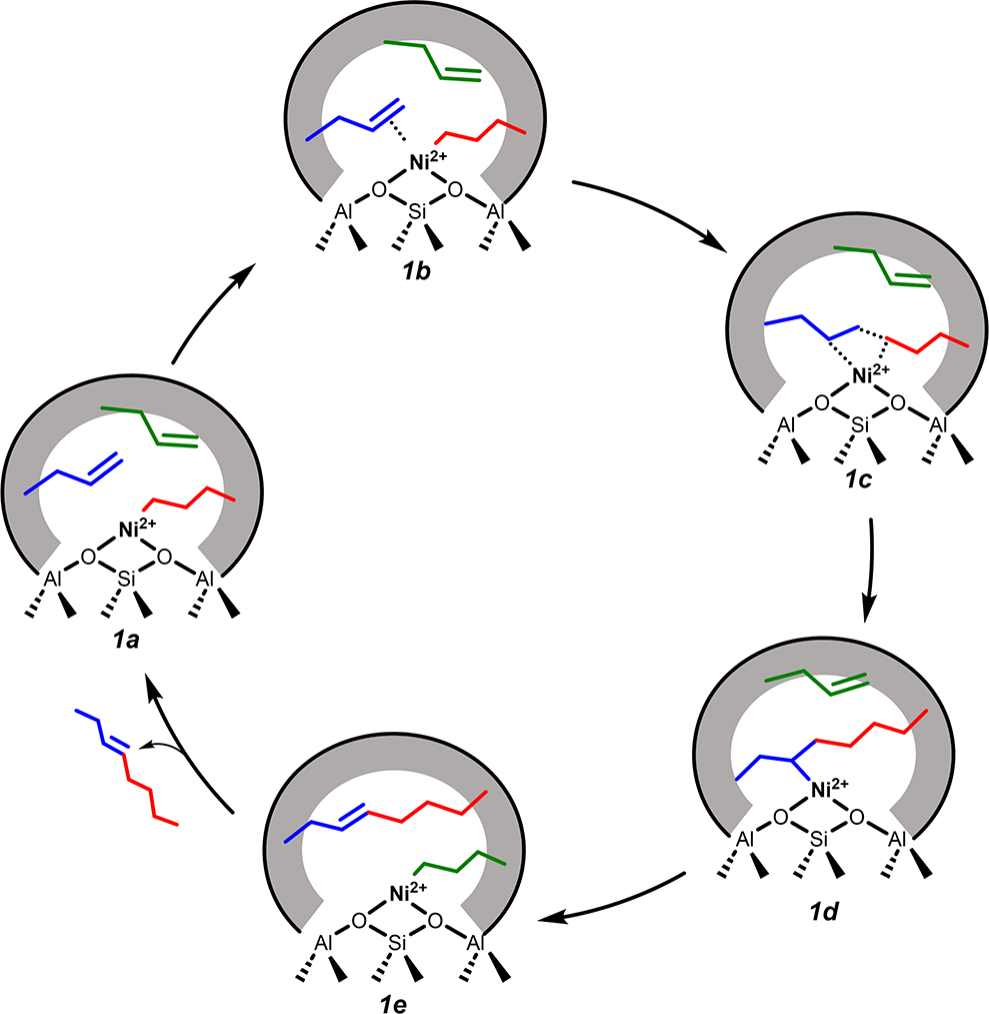
Schematic of the Proposed Reaction Mechanism for 1-Butene Dimerization on Ni^2+^ Sites in Ni-Exchanged Zeolites

The Eley–Rideal (ER)-type C–C bond formation, accompanied by intramolecular H transfer, results in the formation of a Ni-octyl complex (**1d**; Scheme 1). In the proposed ER-type mechanism, the rate of 1-butene conversion must be formally of first order in *p*_butene_, and not between one and two, as observed experimentally. Therefore, we propose that the desorption of the formed octyl complex as an octene is assisted by the adsorption of another 1-butene molecule, subsequently resulting in the formation of the Ni-butyl complex thus completing the catalytic cycle. In other words, the generation of the active site (Ni-butyl complex) is likely also dependent on *p*_butene_, resulting in the observed reaction orders being greater than one.

We must note here in passing that the observed kinetic parameters are also consistent with the previously proposed reaction mechanism proceeding on Ni-butyl complexes in Ni–Ca-LTA zeolites under similar reaction conditions.^8^ In that mechanism, the reaction of two 1-butene molecules on the Ni-butyl complex leads to the formation of the linear octene product. In the first step, two 1-butene molecules adsorb reversibly on the Ni-butyl. The C–C bond formation between the two weakly adsorbed butene molecules is the kinetically rate-determining step, resulting in the observed second-order dependence and the formation of the linear octene product. In the final step, desorption of linear octene regenerates the active site and completes the catalytic cycle. Although it is not possible to distinguish between the two mechanisms based on the present work, we can unequivocally conclude that the rate-determining C–C step must be preceded by an exothermic adsorption of a 1-butene molecule on the active site, resulting in negative values of *E*_A,app_.

### Effect of Local Environment around Ni^2+^ Cations on 1-Butene Dimerization Activity

Under the assumption that the strong exothermic step is the adsorption of 1-butene molecules on the active Ni sites, which precedes the rate-determining C–C coupling step, we can now discuss here qualitatively the trends in the intrinsic activation energy (*E*_A,int_) for 1-butene dimerization on the three zeolite frameworks investigated in this work. Different zeolite frameworks have been shown to have a substantial effect on the adsorption enthalpy (Δ*H*_ads_) of hydrocarbons due to different degree of interaction with the hydrocarbon chains.^53–55^ For example, Eder et al.^53^ experimentally demonstrated that the adsorption enthalpy of linear C_3_–C_6_ alkanes displayed an increment of ∼12 kJ·mol^–1^ per carbon atom on MFI zeolites and ∼7 kJ·mol^–1^ per carbon atom on FAU zeolites. The *y*-intercept was ∼10 kJ·mol^–1^ for both zeolites. Zhao et al.^56^ also observed that the adsorption enthalpy of C_1_–C_4_ linear alcohols on the BAS in MFI zeolite also showed an increment ∼12 kJ·mol^–1^ per carbon atom, respectively, with a y-intercept of ∼75 kJ·mol^–1^. Piccini et al.^45^ and Barrer and Davies^57^ observed that the standard enthalpy of adsorption of C_1_–C_4_ alkanes in CHA zeolites shows an increment of 9–10 kJ·mol^–1^ per carbon atom. Overall, these findings suggest that the adsorption enthalpy of molecules on the active sites in zeolite frameworks is composed of two components: (i) the interaction of the molecule with the active sites (indicated by the *y*-intercept), and (ii) the interaction of the hydrocarbon chain with the zeolite walls (indicated by the linear increment per carbon atom).

Based on thermogravimetric analysis (see Supporting Information Figure S11), we estimated the −Δ*H*_ads_ of 1-butene on a representative Ni-NaMFI zeolite sample to be ∼68 kJ·mol^–1^. For comparison, the −Δ*H*_ads_ of 1-butene on the Ni^2+^ cations in the Ni–Ca-LTA zeolite was estimated to be ∼63 kJ·mol^–1^.^8^ Assuming that the interaction of 1-butene molecules (via their double bonds) is the same for the Ni^2+^ sites in different zeolites, the relative adsorption enthalpies can thus be primarily determined by the interaction of the hydrocarbon chain with the zeolite walls.

Based on the trends reported in the literature (and discussed above), we expect that the magnitude of Δ*H*_ads_ of 1-butene on different zeolite frameworks decreases in the order: |Δ*H*_ads,MFI_| > |Δ*H*_ads,CHA_| > |Δ*H*_ads,FAU_|. In contrast, the magnitude of the apparent negative activation energies on the three zeolite frameworks decreased in the order: |*E*_A,app,FAU_| > |*E*_A,app,CHA_| > |*E*_A,app,CHA_| (see Table 4). Assuming that the transition state is preceded by the exothermic adsorption of the 1-butene molecule on the active site, these trends can only be rationalized, if the intrinsic barrier (*E*_A,int_) for 1-butene dimerization on different zeolite frameworks increases in the order *E*_A,int,FAU_ < *E*_A,int,CHA_ < *E*_A,int,MFI_. Figure 12 illustrates a possible enthalpy diagram for 1-butene dimerization on the active Ni sites that qualitatively describes the predicted trends. This enthalpy diagram is compatible with the reaction mechanism illustrated in Scheme 1 and the overall standard reaction enthalpy of 1-butene dimerization to 1-octene (Δ*H_rxn_*).

**Figure 12:**
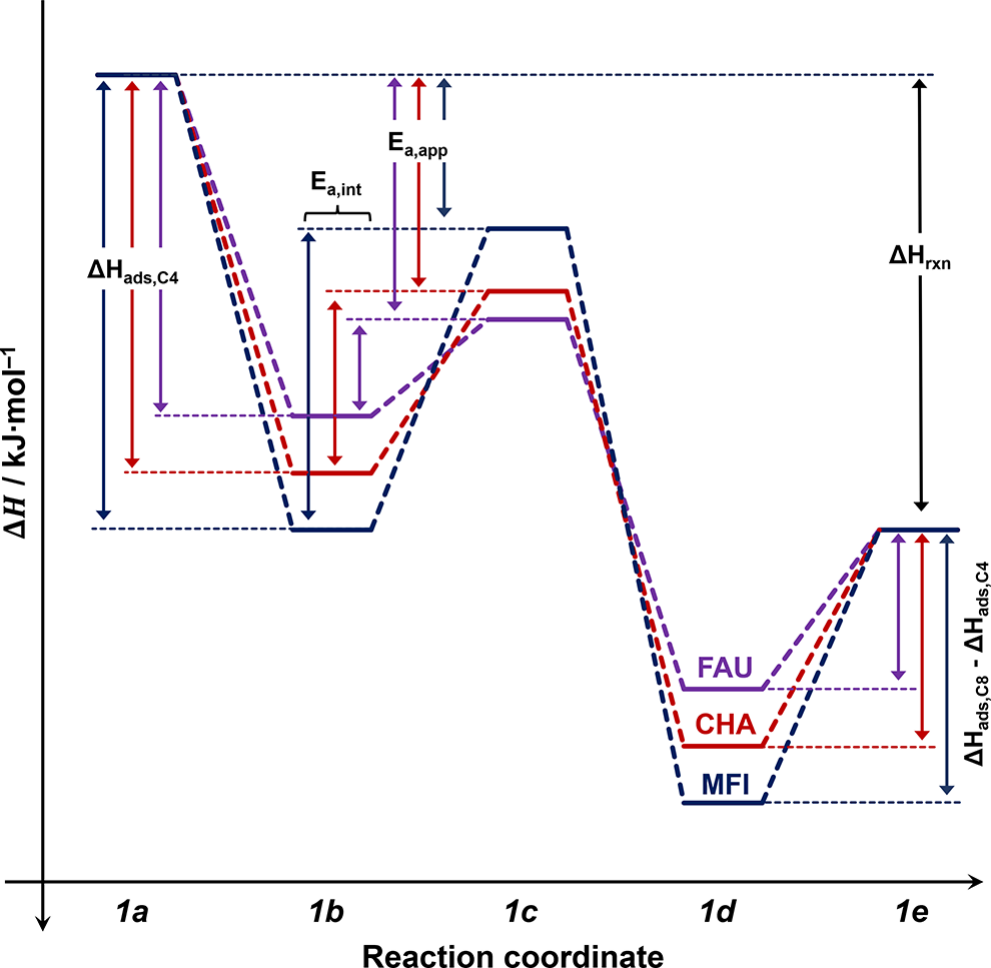
Qualitative enthalpy diagram for initial, transition, and final states illustrating the trends for adsorption enthalpies of 1-butene (Δ*H*_ads_), apparent activation energies (*E*_A,app_), and deduced intrinsic activation energies (*E*_A,int_) for 1-butene dimerization on different zeolite frameworks. The reaction coordinates correspond to the steps described in Scheme 1.

Next, for a (semi-) quantitative analysis, we estimated the intrinsic activation barriers relative to that for 1-butene dimerization on FAU (denoted as Δ*E*_A,int_), based on the experimentally measured apparent activation energies relative to that on FAU (denoted as Δ*E*_A,app_) and the predicted adsorption enthalpies of 1-butene, also relative to that on FAU (denoted as ΔΔ*H*_ads_). Based on the proposed enthalpy diagram, Δ*E*_A,int_ for different zeolite frameworks can be estimated using the following relation

1 $$\Delta E_{A,\mathrm{int}}\approx {\Delta E}_{A,app}-{\Delta \Delta H}_{ads}$$

Based on the literature discussed above, the C_4_ hydrocarbon chains are likely to be stabilized by additional 10 ± 2 kJ·mol^–1^ (2–3 kJ·mol^–1^ per carbon atom) in the cages of the CHA framework compared to the supercages of the FAU framework.^55^ Furthermore, the relatively smaller channel intersections of MFI zeolite additionally stabilize the C_4_ hydrocarbon chains by another 10 ± 2 kJ·mol^–1^ (2–3 kJ·mol^–1^ per carbon atom) compared to CHA.^55^ Based on these trends, the ΔΔ*H*_ads_ values for Ni-NaCHA and Ni-NaMFI zeolites are estimated to be −10 kJ·mol^–1^ and −20 kJ·mol^–1^, respectively (see Table 4). Additionally, based on our kinetic experiments, the Δ*E*_A,app_ for Ni-NaCHA and Ni-NaMFI zeolites is estimated to be +11 kJ·mol^–1^ and +16 kJ·mol^–1^, respectively. From these values, the Δ*E*_A,int_ values are estimated to be +21 kJ·mol^–1^ for Ni-NaCHA and +36 kJ·mol^–1^ for Ni-NaMFI (summarized in Table 4).

Overall, based on the observed trends (see Figure 12) and the Δ*E*_A,int_ values estimated from the (semi-) quantitative analysis (see Table 4), we conclude that the lower intrinsic activation energy for 1-butene dimerization in the FAU zeolites, compared to that in the CHA or MFI zeolites, results in the almost two-orders-of-magnitude higher 1-butene dimerization TOF for Ni-NaFAU zeolite samples. We must also recall here that the TOF_butene_ on the BAS in the FAU framework (∼1.7 mol_butene_·mol_BAS_·s^–1^) was also significantly higher than that on MFI and CHA zeolite frameworks (0.074–0.084 mol_butene_·mol_BAS_·s^–1^).

The diameter of the largest possible sphere that can be included in the three investigated zeolite frameworks follows the order: MFI (∼6.36 Å) < CHA (∼7.37 Å) < FAU (∼11.24 Å).^58^ Therefore, we propose that the size of the cage (or channel intersections) in these zeolites differently stabilizes reactant state, i.e., weakly adsorbed 1-butene molecules and the bulkier C–C coupling transition state. The relatively smaller enclosure around Ni^2+^ cations in the MFI and CHA zeolites stabilizes the adsorbed 1-butene molecules, resulting in relatively higher heat of adsorption but is less suitable to stabilize the large C–C coupling transition state. The FAU framework, on the other hand, with its large supercages, is apt for stabilizing the large C–C coupling transition state, but it provides less stabilization to the reactant state (illustrated in Supporting Information Scheme S1). Overall, these opposite trends manifest in a significantly smaller *E*_A,int_ for 1-butene dimerization in FAU, which in turn results in an almost two orders-of-magnitude higher TOF_butene_. Lastly, we note here in passing that, based on the trends in the predicted Δ*E*_A,int_, we expect the 1-butene dimerization rates in CHA to be higher than those in MFI. However, we speculate that the observed C_8_= products (especially the branched isomers) may be mass transfer-limited due to the smaller pore openings in the CHA framework, leading to relatively lower experimentally observed rates on the Ni-NaCHA zeolites series.

## Conclusions

We have identified isolated Ni^2+^ cations ion-exchanged at Al-pair sites in the CHA, MFI, and FAU zeolite frameworks as the dominating catalytically active sites for 1-butene dimerization through a combination of different characterization techniques (XAS and IR) and activity/selectivity measurements. While a certain product shape selectivity is observed for small-pore zeolite CHA zeolites favoring linear octene formation with high selectivity (∼50% at low 1-butene conversions), the highest rates per Ni are obtained on the Ni-exchanged large-pore FAU zeolites.

The Ni^2+^ ions in FAU are, however, less stabilized and tend to aggregate and form nickel oxide nanoparticles. The formation of nickel oxide nanoparticles even at very low Ni loadings (>50 μmol_Ni_·g_zeolite_^–1^) is attributed to the presence of Na^+^ cations at the preferred S_I_ locations within the zeolite framework. The formation of nickel oxide nanoparticles is prevented in the absence of Na^+^, i.e., by using HFAU zeolites as the precursors. However, only the Ni^2+^ cations that are ion-exchanged at the S_II_ sites of the FAU framework, which are accessible from the supercages, show high activity for 1-butene dimerization under supercritical reaction conditions.

We propose that the 1-butene dimerization in Ni-exchanged CHA, MFI, and FAU zeolites follows a Cossee–Arlman-type coordination-insertion mechanism, propagating on isolated Ni sites. Based on the obtained reaction orders and the negative apparent activation energies, the rate-determining C–C bond formation step is preceded by the strong adsorption of reactants on the active site.

For samples containing predominantly exchanged Ni cation sites, we estimated the 1-butene dimerization turnover frequency (TOF_butene_) of these sites. The Ni-sites in FAU convert 1-butene at rates almost two-orders-of-magnitude greater than the Ni-sites in MFI and CHA zeolites. This difference is attributed to a smaller intrinsic activation energy for C–C coupling in FAU, as a result of a more spacious environment in its supercages, which does not sufficiently stabilize the adsorbed state of 1-butene molecules while stabilizing the bulkier C–C coupling transition state in comparison to the more-constrained CHA and MFI frameworks.

## Experimental Section

### General Information

Sodium acetate (NaOAc; ≥99% purity), nickel acetate (Ni­(OAc)_2_; ≥99% purity), and cobalt nitrate (Co­(NO_3_)_2_; ≥98% purity) were purchased from Sigma-Aldrich and used without further purification. Deionized (DI) water was used to prepare all of the aqueous solutions.

### Catalyst Synthesis

Two parent CHA zeolites with similar Al content (Si/Al ∼11) but different concentrations of Al pair sites were synthesized according to a modified recipe from literature.^59,60^ Refer to Section S1 of the Supporting Information for a detailed synthesis procedure. A third commercially available CHA zeolite sample was acquired from Clariant (H-SSZ-13; Si/Al ∼ 16). Parent MFI zeolite (NH_4_-ZSM-5; Si/Al ∼ 15) and parent FAU zeolite (CBV 720, Si/Al ≈ 15) were purchased from Zeolyst International. All zeolite samples were calcined in flowing synthetic air (20 vol % O_2_/N_2_; ∼100 mL·min^–1^) for 6 h at 823 K (heating rate: 3 K·min^–1^) to obtain the clean H-form of the zeolites.

### Ion Exchange with Na^+^ and Ni^2+^ Cations

Na-form of the zeolites was prepared by three ion exchanges of the H-form with 0.06 M NaOAc (∼20 g_solution_·g_zeolite_^–1^) overnight at 353 K. After each ion exchange, the solid was washed thoroughly with DI water (∼20 g_water_·g_zeolite_^–1^) and dried at 353 K for several hours. After the final ion exchange and a calcination procedure in flowing synthetic air (∼100 mL·min^–1^) for 6 h at 773 K (heating rate: 3 K·min^–1^), the zeolite was washed twice with 0.1 M NaOAc (∼20 g_solution_·g_zeolite_^–1^) and centrifuged (for 3 min at 4000 rpm) to separate the powder. The zeolite was calcined in flowing synthetic air (∼100 mL·min^–1^) for 6 h at 773 K (heating rate: 3 K·min^–1^), resulting in the Na-form.

The Ni^2+^ introduction was carried out as a Ni–Na coexchange with varying concentrations of the exchange solutions (∼20 g_solution_·g_zeolite_^–1^) overnight at 353 K. The concentration of the solutions varied between 0.002 and 0.06 M Ni­(OAc)_2_ and 0.001–0.6 M NaOAc to obtain loadings between 25 and 1100 μmol_Ni_·g_zeolite_^–1^. The pH varied between 6 and 7 during the ion-exchange procedure. After ion exchange, the solid was washed thoroughly with DI water, dried at 353 K for several hours, and calcined in flowing synthetic air (∼100 mL·min^–1^) for 6 h at 773 K (temperature ramp: 3 K·min^–1^).

The Na- and Ni-exchanged CHA zeolite samples are referred to as Ni-NaZ­(X), where “Z” denotes the zeolite framework (CHA, MFI, or FAU) and “X” refers to the Ni content in the zeolites, expressed in μmol_Ni_ g_zeolite_^–1^.

The H-form of the parent FAU zeolite was also ion exchanged directly (i.e., without prior Na^+^ exchange) with Ni^2+^ at varying Ni loadings (ranging between 30 and 300 μmol_Ni_ g_zeolite_^–1^) by using the same ion-exchange procedure. These zeolite samples are termed Ni-HFAU­(X), where “X” is the Ni content in μmol_Ni_ g_zeolite_^–1^.

### Catalyst Characterization

The Si-, Al-, Na-, Ni-, and Co-contents in the zeolite samples were determined by AAS conducted using a Thermo Fisher Solar M5 Dual Flame graphite furnace atomic absorption spectrometer. After the samples were dried at 523 K for 24 h, they were dissolved in a mixture of HF and HNO_3_ and injected into the graphite furnace. The concentration of each element was determined via previously performed calibration for each element.

The adsorption of 1-butene was measured thermogravimetrically on a microbalance in a Seteram TG-DSC 111 calorimeter connected to a high vacuum system. For this, ∼25 mg of the sample was pretreated at 723 K for 1 h under vacuum (<10^–4^ mbar) and then cooled to 313 K. During the experiments, 1-butene was introduced into the system in small dosing steps, resulting in equilibrium pressure ranging between 0.003 and 500 mbar. The butene uptake was determined by the increase in sample weight, and the released heat was monitored by the heat flux signal.

XRD patterns were recorded under ambient conditions on a PANalytical Empyreal System diffractometer with a Cu Kα radiation source (λ = 1.54 Å) operating at 45 kV and 40 mA. A sample spinner was utilized to record XRD patterns in the range of 5–50° with a step size of 0.013°.

N_2_ physisorption was performed at a liquid N_2_ temperature on a PMI Automatic Sorptometer. The samples were evacuated at 523 K for 2 h (heating rate: 5 K·min^–1^) prior to the measurement.

Scanning electron microscopy (SEM) images were collected on a JEOL JSM 7500F instrument.

### Determination of the Al Pair Concentration

The Al pair concentration was determined according to the ion-exchange procedure developed by Dědeček et al.^61^ For this, the Na-form of the zeolite was stirred overnight in 0.05 M Co­(NO_3_)_2_ (∼150 mL_solution_·g_zeolite_^–1^) under ambient conditions. The sample was washed three times with DI water and then dried in an oven at 353 K for several hours. This exchange was performed three times in total. The solid was finally calcined in flowing synthetic air (∼100 mL·min^–1^) at 773 K for 6 h (heating rate: 3 K·min^–1^). The Co content was subsequently determined by AAS to reveal the number of Al pairs in the zeolite samples.

### IR Spectroscopy Measurements

BAS and Lewis acid site (LAS) concentrations were determined via pyridine adsorption and monitored by IR spectroscopy. For this, the samples were pressed into self-supporting wafers (ρ_A_ ≈ 10 mg·cm^–2^), which were inserted into the measuring cell of a Nicolet 5700 FT-IR spectrometer (Thermo Electron Corporation) equipped with a liquid N_2_ cooled detector. Prior to measurements, the sample was activated for 1 h at 723 K under vacuum (<10^–5^ mbar, heating rate: 10 K·min^–1^). After cooling to 423 K, pyridine was equilibrated at 0.5 mbar for at least 1 h. The system was then evacuated for 1 h prior to the measurements. The spectra were measured after activation and outgassing in the range from 400 to 4000 cm^–1^ (120 scans, resolution: 2 cm^–1^). The difference spectra were analyzed according to the characteristic bands for BAS at ∼1540 cm^–1^ and LAS at ∼1450 cm^–1^. The respective acid site concentrations (*C*_acid_) were estimated using the following equation

2 $$C_{acid}=\frac{A_{\mathrm{int}}\cdot A_{wafer}}{m_{wafer}\cdot \epsilon }$$

where *A*_int_ denotes the integrated peak area, *A*_wafer_ is the area of the wafer, *m*_wafer_ is the mass of the wafer, and ϵ denotes the respective extinction coefficients (equal to 0.73 cm^–1^·mol^–1^ for BAS and 0.96 cm^–1^·mol^–1^ for LAS).^62,63^

Low-temperature CO adsorption was conducted on a Vertex 70 spectrometer by Bruker Optics equipped with a liquid N_2_ cooled detector. A self-supporting wafer was prepared as described above and activated in the measurement cell for 1 h at 723 K under vacuum (<10^–5^ mbar, heating rate: 10 K·min^–1^). Synthetic air (∼25 mbar) was dosed into the cell for another hour. The temperature was then reduced to 373 K. Next, the cell was evacuated (*p* < 10^–6^ mbar) and further cooled to liquid N_2_ temperature. The adsorption of CO was performed by dosing with systematically increasing pressure steps, ranging between 0.1 μbar and 1 mbar. The next step was initiated only after stabilization of the spectral features. Scans were regularly taken in the range from 1250 to 4000 cm^–1^ (120 scans with a resolution of 2 cm^–1^).

### XAS Measurements

Ni K-edge (8333 eV) X-ray absorption spectra on Ni-NaMFI samples were obtained at the P65 beamline of the German electron synchrotron (DESY) in Hamburg, Germany. Ni K-edge XANES spectra for the activated Ni-NaFAU and Ni-HFAU zeolite samples were measured at the Balder beamline of the Max IV synchrotron radiation facility in Lund, Sweden. The Ni K-edge EXAFS measurements on the Ni-NaFAU and Ni-HFAU zeolite samples were performed at the NOTOS beamline of the ALBA synchrotron facility in Barcelona, Spain. Refer to the Supporting Information Section S1 for experimental details.

### Butene Dimerization Reaction

The catalytic performance of the catalysts for the butene 1-dimerization reaction was analyzed in a fixed bed plug flow reactor (ϕ_i.d._ = 3.9 mm). The reaction feed (85% 1-butene and 15% isobutane) was introduced using a syringe pump (ISCO model 500 D). Isobutane is inert under the investigated reaction conditions and was used as an internal standard for normalization of areas measured by gas chromatography (GC). The temperatures of the reactor and the tubing were controlled by an Eurotherm temperature controller. The temperature of the tubing was kept constant at approximately 423 K. The reaction pressure was maintained and regulated by a Tescom back-pressure regulator. The gas stream was hydrogenated with H_2_ on a Pt/Al_2_O_3_ catalyst prior to the analysis. An online Agilent HP 7890 GC, equipped with a 50 m HP-1 column and flame ionization detector, was used for product analysis.

Prior to being weighed, the catalyst was dried at ∼373 K for at least 1 h. The catalyst bed was diluted with SiC (with catalyst/SiC ratio ∼ 1:10) and placed in the isothermal zone of the reactor between two quartz wool plugs. After activation in synthetic air for 2 h at 723 K (temperature ramp: 10 K·min^–1^), the reactor was cooled down to the reaction temperature, while the bypass was flushed with the feed. Next, the desired flow rate was set, and after at least three stable GC measurements of the bypass, the flow was redirected into the reactor and periodic product analysis using the GC was started.

Standard measurements were performed at ∼433 K and ∼50 bar total pressure with a feed flow rate typically varying between 0.04 and 0.24 mL·min^–1^. Catalyst loading was varied between 1 and 200 mg. In some cases, the catalyst was diluted with SiO_2_ in a 1:10 weight ratio. The weight-normalized space-velocity was in the range of 6–4000 g_butene_ g_zeolite_^–1^ h^–1^ or 0.03–20 mol_butene_ g_zeolite_^–1^ s^–1^.

The activation energies were determined from Arrhenius plots obtained at temperatures ranging from 423 to 453 K at *p*_total_ ≈ 50 bar. The reaction orders in *p*_butene_ were determined at 423 K and *p*_total_ varying between 40 and 55 bar.

Conversion (*X*) and selectivity (*S*) were calculated according to the following equations

3 $$X_{butene}=\frac{{\left(n_{butene}\right)}_{in}-{\left(n_{butene}\right)}_{out}}{{\left(n_{butene}\right)}_{in}}$$

4 $$S_{product}=\frac{{\left(n_{product}\right)}_{out}}{{\left(n_{butene}\right)}_{in}-{\left(n_{butene}\right)}_{out}}\cdot \frac{\left|\nu_{butene}\right|}{\nu_{product}}$$

where *n*_butene_ and *n*_product_ are the number of moles of butene and the product, respectively, while ν_butene_ and ν_product_ are the stoichiometric coefficients of butene and the product in a balanced equation, respectively.

External mass transport limitations were excluded by performing the reactions at varying flow rates (0.04–0.24 mL min^–1^) but at constant weight-normalized space-velocity (see Supporting Information Figure S12). Exemplary conversion versus time-on-stream plots for representative catalysts samples are presented in Supporting Information Figure S13. For the catalysts showing significant deactivation, the 1-butene conversion rate was evaluated at short times-on-stream values.

## Supplementary Material



## Notes

Additional experimental details, additional physicochemical characterization, additional IR spectroscopy results, elemental analysis results, and additional XAS results (PDF)
