# Supplementary material for: Isolated Ni2+ Cations as the Active Centers for 1‑Butene Dimerization in Zeolites
Source: JACS Au. 2025 Jun 30;5(7):3350–65. doi: 10.1021/jacsau.5c00461 (PMC12308395; doi:10.1021/jacsau.5c00461)
Supplement: Supplementary file 1 [file au5c00461_si_001.pdf]

Supporting Information for

Isolated Ni^{2+} Cations as the Active Centers for 1-Butene Dimerization in Zeolites

Laura Löbbert[a], Abelina Ellert[a], Mengjie Zhou[a], Ricardo Bermejo-Deval[a], Noelia Barrabes[b], Rachit Khare[a]*, Maricruz Sanchez-Sanchez[a,c]* and Johannes A. Lercher[a,d]*

[a]Department of Chemistry and Catalysis Research Center, Technical University of Munich, Garching, 85748, Germany

[b]Institute of Material Chemistry, Vienna University of Technology, Vienna, A-1060, Austria

[c]Institute of Chemical Environmental and Bioscience Engineering, Vienna University of Technology, Vienna, 1060, Austria

[d]Institute for Integrated Catalysis, Pacific Northwest National Laboratory, Richland, Washington, 99354, United States

*Corresponding authors: rachit.khare@tum.de, maricruz.sanchez@tuwien.ac.at, johannes.lercher@ch.tum.de

Table of Contents

S1. Additional Experimental Details 3

S2. Physicochemical Characterization of CHA Zeolites 6

S3. Physicochemical Characterization of MFI Zeolites 13

S4. Physicochemical Characterization of FAU Zeolites 20

S5. Additional Figures 24

References 30

S1. Additional Experimental Details

S1.1. Synthesis of CHA zeolite samples

The CHA zeolites were synthesized according to a modified recipe from literature.[1, 2] In a typical synthesis of the CHA with ~50 % Al pairs, 28.1 g of an aqueous N,N,N-trimethyl-1-adamantyl-ammonium hydroxide (TMAdaOH) solution (26.6 mmol, 20 wt.%, Sachem) were mixed with 25.7 g of deionized (DI) water (18.2 MΩ·cm, 1425 mmol) and stirred for 15 min at room temperature. Then, 0.354 g of aluminum hydroxide hydrate ($Al(OH)_3 \cdot xH_2O$, ~3.69 mmol, ≥98 %, Sigma-Aldrich) was added and the mixture was thoroughly stirred for another 15 min. After adding 10 g of silica (Ludox, 66.6 mmol, 40 wt.%, Sigma-Aldrich) the contents were homogenized under ambient conditions for 2 h. The synthesis gel resulted in a molar ratio of 6.67 SiO_2:0.369 $Al(OH)_3$:2.66 TMAdaOH:3007 H_2O_{total}. The gel was transferred to a 100 mL Teflon-lined stainless-steel autoclave (Toption Instruments) and heated in a forced convection oven at 433 K for 6 days under rotation of 30 rpm.

In a typical synthesis of the CHA with ~95 % Al pairs, 26.6 g of aqueous TMAdaOH solution (25.1 mmol) were mixed with 24.3 g of DI water (1347 mmol) and stirred for 15 min under ambient conditions. Then, 0.327 g of $Al(OH)_3 \cdot xH_2O$ (~3.41 mmol) and 3.85 g of an aqueous 5 M NaOH solution (16.1 mmol, ≥98 %, Sigma-Aldrich) were added. After each addition, the mixture was thoroughly stirred for 15 min. Next, 9.45 g of silica (62.9 mmol) were added, and the contents were homogenized under ambient conditions for 2 h. The synthesis gel resulted in a molar ratio of 6.29 SiO_2:0.341 $Al(OH)_3$:2.51 TMAdaOH:3021 H_2O_{total}. The gel was transferred to a 100 mL Teflon-lined stainless-steel autoclave (Toption Instruments) and heated in a forced convection oven at 433 K for 6 days under rotation of 30 rpm.

The resulting solid CHA products were washed thoroughly with DI water and acetone in alternating steps until the pH stayed constant at around 6. After the final washing step with water, the product was fully dried in the oven at 353 K and then calcined in flowing synthetic air (20 vol.% O_2/N_2, ~100 $mL \cdot min^{-1}$) at 853 K for 10 h (heating ramp: 1 $K \cdot min^{-1}$).

S1.2. X-ray absorption spectroscopy measurements

Ni K-edge (8333 eV) X-ray absorption spectroscopy (XAS) measurements on Ni-NaMFI samples were carried out at the P65 beamline of the German synchrotron facility (DESY) in Hamburg, Germany.[3] The PETRA III storage ring operated at 6 GeV energy and 100 mA beam-current in top-up mode. A water-cooled Si111 double crystal monochromator (DCM)

was used for obtaining monochromatic X-rays. Two Si mirrors were installed in front of the DCM to reject higher harmonics. The DCM was calibrated for Ni K-edge by measuring a Ni-foil and defining the first major inflection point as 8333 eV. A Ni-foil was also placed between the second and third ionization chamber for the energy calibration of each measured spectrum. The XAS spectra were measured in both transmission mode and in fluorescence mode using a passivated implanted planar silicon (PIPS) detector.

In situ measurements were performed using a quartz capillary micro-reactor setup. In a typical experiment, the catalyst was placed in a quartz capillary ($\phi_{o.d.}$ = 1 mm, 20 μm thickness) supported between two quartz wool plugs. The capillary was heated from below with a hot-air gas-blower (Oxford FMB). Gas flow rates were maintained using Bronkhorst electronic mass flow controllers and the pressure was continuously monitored using a pressure gauge (Omega). The catalyst sample was first activated under 5 mL·min^{-1} O_2 (10 vol.% in He) at 723 K for 1 h (heating ramp: 10 K·min^{-1}). After activation, the sample was cooled down to 433 K for XAS measurements. Spectra were also measured on the activated sample under a flow of 5 mL·min^{-1} 1-butene at 433 K and ambient pressure.

Ni K-edge X-ray absorption spectra of activated Ni-HFAU and Ni-NaFAU zeolite samples were measured at the Balder beamline of the Max IV synchrotron radiation facility in Lund, Sweden. The ~3 GeV storage ring operated at ~500 mA current in top-up mode. A liquid N_2-cooled Si111 DCM was used for obtaining monochromatic X-rays and a Si-coated plane mirror was used for rejecting higher harmonics. All spectra were measured in total fluorescence yield (TFY) mode using a PIPS detector.

In situ measurements were performed using the quartz capillary micro-reactor setup described above. In a typical experiment, the catalyst sample was first activated under 5 mL·min^{-1} O_2 (10 vol.% in He) at 723 K for 2 h (heating ramp: 10 K·min^{-1}). After activation, the sample was cooled down to room temperature for XAS measurements. Spectra were also measured on the activated sample under 1 bar ethene atmosphere at room temperature and ambient pressure.

Lastly, XAS measurements on the as-synthesized Ni-HFAU and Ni-NaFAU zeolite samples were performed at the NOTOS Beamline of the ALBA synchrotron facility in Barcelona, Spain. All spectra were measured in fluorescence mode in the beamline's sample holder for static measurements. For this, the catalysts were pressed into pellets and secured by a Kapton tape.

The data were monitored for any signs of X-ray beam damage. Several successive scans were averaged to reduce signal-to-noise ratio and improve the data quality. For XANES analyses, E_0 was fixed at 8333 eV and the spectra were normalized and flattened. For EXAFS analyses, spectra were background subtracted, normalized, k^2-weighted, and Fourier-transformed in the k range of 2.4 – 12 $Å^{-1}$. The EXAFS fitting was performed in k-space on the k^1-, k^2-, and k^3-weighted data. A Ni-foil was fitted to obtain the amplitude reduction factor, $S_0^2 = 0.8$, which was then used in the subsequent fits. XANES and EXAFS data analyses were performed using Athena and Artemis software packages.[4]

S2. Physicochemical Characterization of CHA Zeolites

X-ray diffraction (XRD) patterns and N_2 physisorption experiments revealed the preservation of the zeolite framework throughout the ion exchange for all three CHA materials (see **Figure S1(a,b)**, **Figure S2(a,b)** and **Figure S3(a,b)**). The introduction of cations did not reduce the crystallinity, the micropore volume or the BET surface area significantly. Only the CHA_H sample showed minor reduction in the specific surface area.

The acidity of the CHA catalysts was analyzed by IR spectroscopy measurements. As pyridine is too large to penetrate the small 8MR channels of the CHA, it was not possible to determine the BAS concentrations quantitatively. The IR spectra in **Figure S1(c)**, **Figure S2(c)** and **Figure S3(c)** show the reduction of BAS by the decrease of the 3612 cm^{-1} vibration upon cation introduction. It can be observed that the –OH vibration of BAS slightly increases again after Ni introduction. The co-introduction of BAS during the Ni exchange was observed for all here reported zeolites and can be easily monitored in the IR spectra of the synthesized CHA zeolites (**Figure S2(c)** and **Figure S3(c)**). Small remaining BAS concentration in the Ni-NaCHA samples, therefore, cannot be excluded.

The morphology of the CHA samples was investigated by SEM (**Figure S1(d)**, **Figure S2(d)** and **Figure S3(d)**). The crystallites of the CHA_L and CHA_H are shaped in the form of cubes while the CHA_M zeolite features sphere-shaped crystals. The crystallite size for all three CHA zeolites is estimated to be approximately 1 – 2 μm.

Tables S1–3 summarize the Na and Ni concentrations in different Ni-NaCHA zeolite samples investigated in this work.

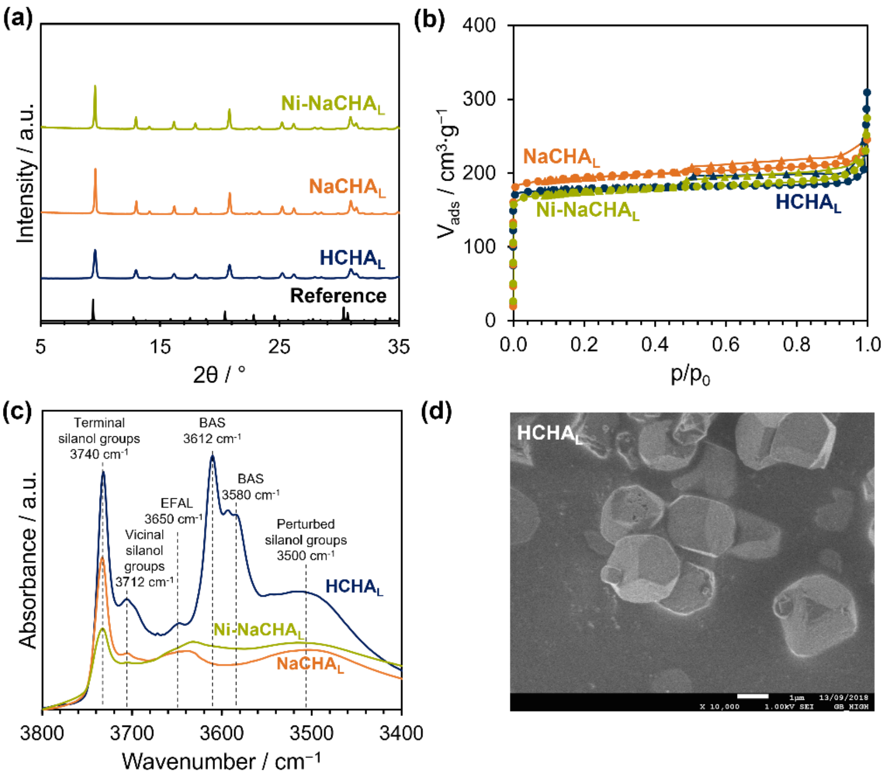

Figure S1. (a) XRD patterns, (b) N_2 physisorption isotherms and (c) IR spectra of $HCHA_L$ (blue), $NaCHA_L$ (orange) and Ni-$NaCHA_L$ (green) zeolite samples. (d) SEM micrograph of the $HCHA_L$ zeolite sample.

Table S1. Ni and Na contents in different Ni-NaCHA$_L$ zeolite samples investigated in this work.

**Sample**	**Ni content /μmol$_{Ni}$·g$_{zeolite}^{-1}$**	**Na content /μmol$_{Na}$·g$_{zeolite}^{-1}$**
Ni-NaCHA$_L$(46)	46	796
Ni-NaCHA$_L$(96)	96	735
Ni-NaCHA$_L$(174)	174	705
Ni-NaCHA$_L$(261)	261	792
Ni-NaCHA$_L$(467)	467	565
Ni-NaCHA$_L$(535)	535	613
Ni-NaCHA$_L$(884)	884	687
Ni-NaCHA$_L$(1039)	1039	600

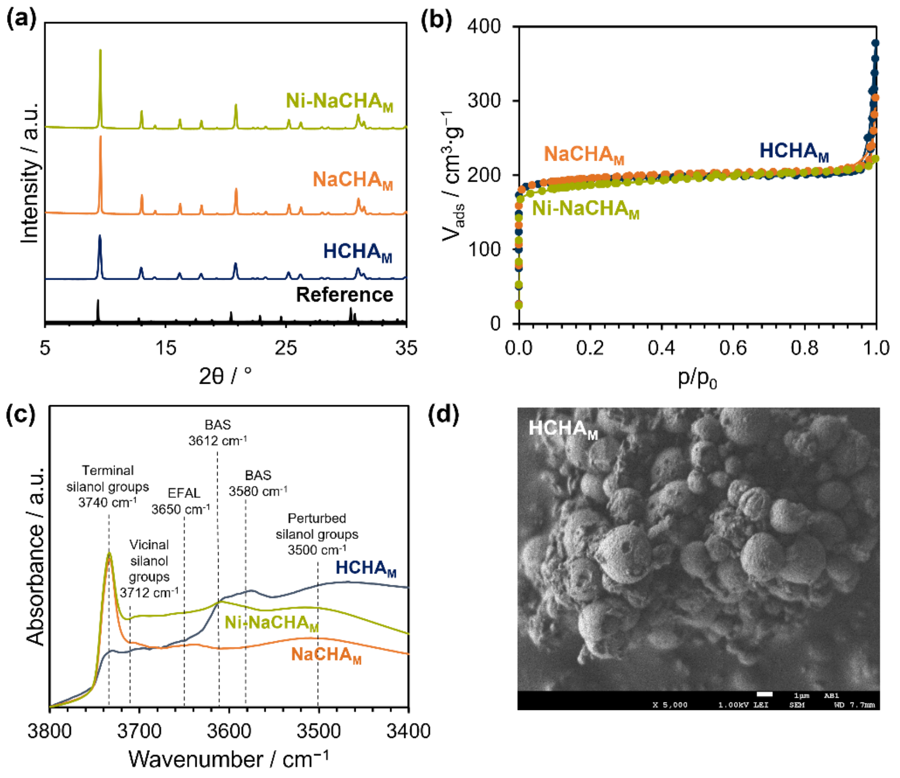

Figure S2. (a) XRD patterns, (b) N_2 physisorption isotherms and (c) IR spectra of HCHA$_M$ (blue), NaCHA$_M$ (orange) and Ni-NaCHA$_M$ (green) zeolite samples. (d) SEM micrograph of the HCHA$_M$ zeolite sample.

Table S2. Ni and Na contents in different Ni-NaCHA$_M$ zeolite samples investigated in this work.

**Sample**	**Ni content /$\mu mol_{Ni} \cdot g_{zeolite}^{-1}$**	**Na content /$\mu mol_{Na} \cdot g_{zeolite}^{-1}$**
Ni-NaCHA$_M$(62)	62	1131
Ni-NaCHA$_M$(93)	93	918
Ni-NaCHA$_M$(250)	250	1005
Ni-NaCHA$_M$(458)	458	1005
Ni-NaCHA$_M$(746)	746	1018

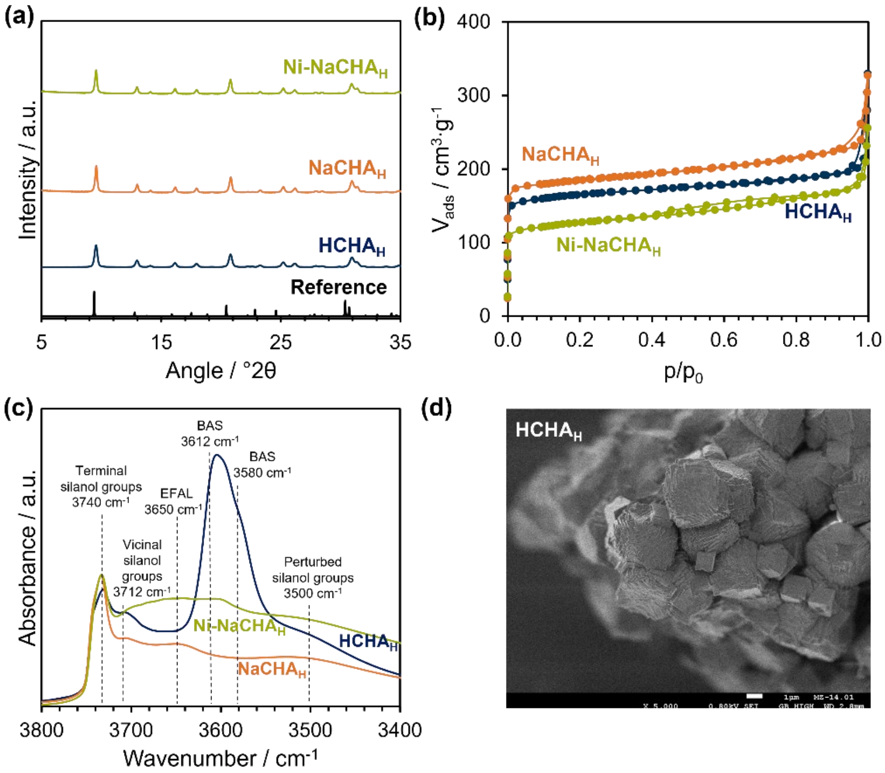

Figure S3. (a) XRD patterns, (b) N_2 physisorption isotherms and (c) IR spectra of $HCHA_H$ (blue), $NaCHA_H$ (orange) and $Ni\text{-}NaCHA_H$ (green) zeolite samples. (d) SEM micrograph of the $HCHA_H$ zeolite sample.

Table S3. Ni and Na contents in different Ni-NaCHA$_H$ zeolite samples investigated in this work.

**Sample**	**Ni content /$\mu mol_{Ni} \cdot g_{zeolite}^{-1}$**	**Na content /$\mu mol_{Na} \cdot g_{zeolite}^{-1}$**
Ni-NaCHA$_H$(64)	64	1418
Ni-NaCHA$_H$(116)	116	1314
Ni-NaCHA$_H$(174)	174	1274
Ni-NaCHA$_H$(356)	356	1087
Ni-NaCHA$_H$(659)	659	618

S3. Physicochemical Characterization of MFI Zeolites

XRD and SEM demonstrated the preservation of the zeolite framework throughout the ion exchange procedure (see **Figure S4(a,d)**). The Brønsted and Lewis acid site concentrations of Ni-NaMFI zeolite samples were determined by IR spectroscopy of adsorbed pyridine, according to the characteristic bands for Brønsted acidity at 1540 cm^{-1} and Lewis acidity at 1450 cm^{-1} (see **Figure S4(c)**). **Table S4** summarizes the BAS and LAS concentrations for different MFI samples investigated in this work.

N_2 sorption experiments showed a decrease of the BET surface area as well as the micropore volume with increasing Ni loadings (see **Figure S4(b)** and **Table S5**) leading to the assumption that particles form within the pores of MFI.

Next, we also examined the nature of active Ni sites in different Ni-NaMFI zeolite samples using IR spectroscopy of adsorbed CO. The FTIR spectra of Ni-NaMFI zeolites at different CO partial pressures ($p_{CO} \approx 0.0001 - 1$ mbar) are presented in **Figure S5**. The vibration band at ~2212 cm^{-1} is attributed to CO adsorbed on the isolated Ni^{2+} cations located at the ion exchange sites in the MFI zeolite (denoted as Ni^{2+}–CO).[5-7] A shoulder to this band, at ~2204 cm^{-1}, is likely associated with dicarbonyl species (Ni^{2+}–$(CO)_2$), also adsorbed on the isolated Ni^{2+} cations.[5] The band at ~2179 cm^{-1} is assigned to CO interacting with Na^+ cations or the BAS in the zeolite framework (Na^+/BAS–CO).[5, 6, 8, 9] Notably, this band appeared only at relatively high CO partial pressures ($p_{CO} > 0.05$ mbar). Additionally, in the Ni-NaMFI samples with relatively high Ni loadings (*i.e.*, >270 $\mu mol_{Ni} \cdot g_{zeolite}^{-1}$), a shoulder at ~2194 cm^{-1} became apparent, which is assigned to CO adsorbed on Ni^{2+} grafted onto the external surface,[10] or on Ni^{2+} within the NiO_x nanoparticles (denoted as NiO_x–CO).[6] In agreement with the rate versus Ni loading trend, the appearance of this band suggests the formation of NiO_x clusters in samples with Ni loading > 270 $\mu mol_{Ni} \cdot g_{zeolite}^{-1}$. Lastly, at CO partial pressures >0.05 mbar, additional bands corresponding to CO adsorbed on (i) Ni^+-monocarbonyl (Ni^+–CO) at ~2111 cm^{-1}, (ii) Ni^+-dicarbonyl (Ni–$(CO)_2$) at ~2130 cm^{-1}, and (iii) Ni^0-polycarbonyl species (Ni^0–$(CO)_x$) at ~2070 cm^{-1}, were evident.[6, 7, 10] The appearance of these bands indicates the partial reduction of Ni^{2+} cations to Ni^+ and Ni^0 at relatively high CO partial pressures.

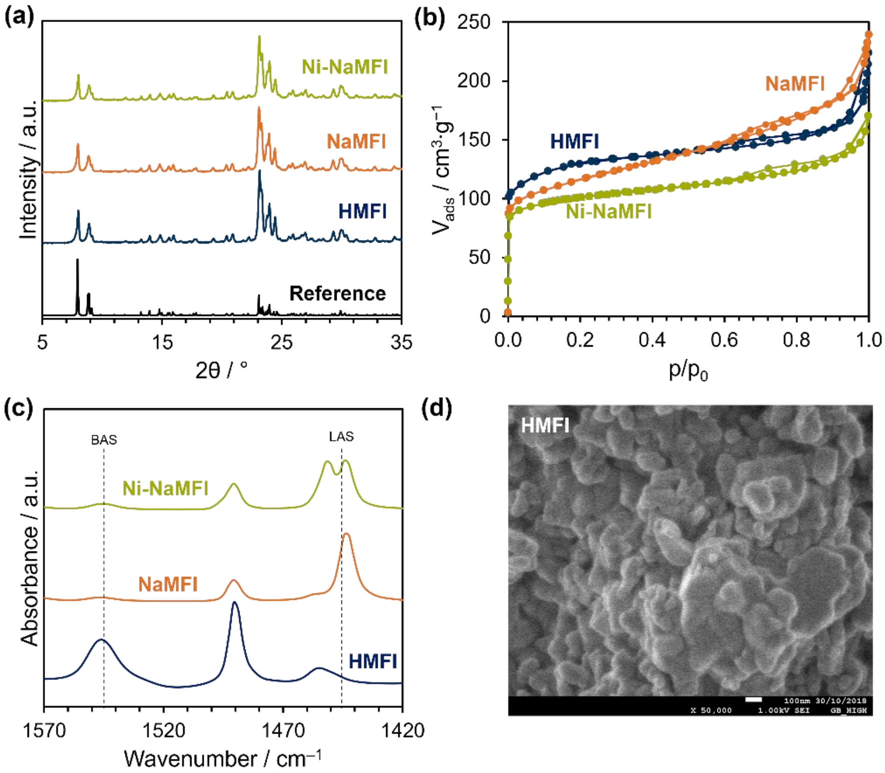

Figure S4. (a) XRD patterns, (b) N_2 physisorption isotherms, and (c) IR spectra of HMFI (blue), NaMFI (orange) and Ni-NaMFI (green) zeolite samples. (d) SEM micrograph of the HMFI zeolite sample.

Table S4. Na, Ni, BAS and LAS concentration for HMFI, NaMFI and Ni-NaMFI zeolite samples investigated in this work.

**Sample**	**Na** **/μmol$_{Na}$·g$_{zeolite}$$^{-1}$**	**Ni** **/μmol$_{Ni}$·g$_{zeolite}$$^{-1}$**	**BAS** **/μmol$_{BAS}$·g$_{zeolite}$$^{-1}$**	**LAS** **/μmol$_{LAS}$·g$_{zeolite}$$^{-1}$**
HMFI	-	-	957	153
NaMFI	970	0	8	810
Ni-NaMFI(52)	718	52	134	998
Ni-NaMFI(85)	713	85	21	181
Ni-NaMFI(126)	696	126	13	154
Ni-NaMFI(170)	705	170	44	447
Ni-NaMFI(179)	613	179	89	923
Ni-NaMFI(206)	626	206	43	373
Ni-NaMFI(298)	622	298	58	468
Ni-NaMFI(496)	692	496	116	428
Ni-NaMFI(671)	713	671	60	983

Table S5. BET specific surface area, micropore volume and mesoporous surface area determined by N_2 sorption experiments for different MFI zeolite samples.

Sample	BET surface area $/m^2 \cdot g_{zeolite}^{-1}$	Micropore volume $/cm^3 \cdot g_{zeolite}^{-1}$	Mesoporous surface area $/m^2 \cdot g_{zeolite}^{-1}$
HMFI	402	0.18	49.1
NaMFI	375	0.12	140
Ni-NaMFI(85)	338	0.13	65.8
Ni-NaMFI(179)	337	0.14	55.6
Ni-NaMFI(496)	313	0.12	74.2
Ni-NaMFI(671)	330	0.10	132

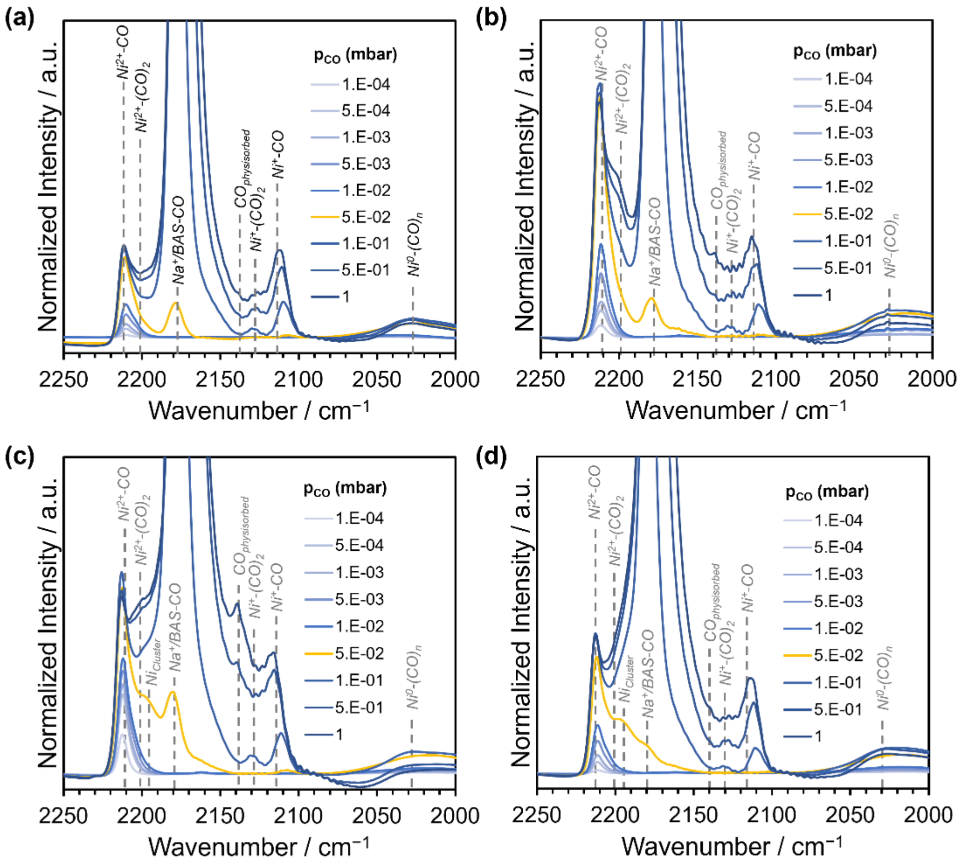

Figure S5. Normalized IR difference spectra of (a) Ni-NaMFI(85), (b) Ni-NaMFI(179), (c) Ni-NaMFI(496) and (d) Ni-NaMFI(671) zeolite samples at different CO partial pressures (p_{CO}). Pressure during dosing steps from $1 \cdot 10^{-4}$ mbar (light blue) to 1 mbar (dark blue) levels are given in mbar. Spectra at $5 \cdot 10^{-2}$ mbar are highlighted in yellow. Dosing of CO was performed at liquid N_2 temperature. The spectra were normalized to zeolite lattice vibrations at approximately between 2100 cm^{-1} and 1700 cm^{-1}.

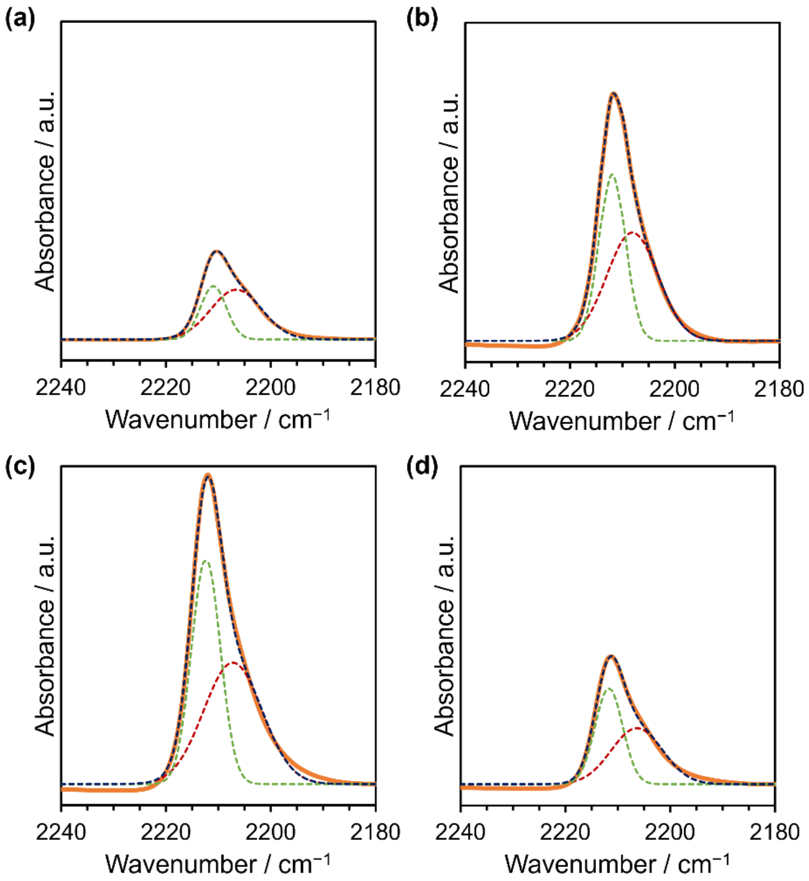

Figure S6. Deconvoluted and normalized IR difference spectra for low-temperature CO adsorption (at $p_{CO} = 1 \cdot 10^{-2}$ mbar) on (a) Ni-NaMFI(85), (b) Ni-NaMFI(179), (c) Ni-NaMFI(496) and (d) Ni-NaMFI(671) zeolite samples. CO dosing was performed at liquid N_2 temperature. Two Gaussian peaks were fitted with a fixed baseline applying Levenberg-Marquardt iteration mechanism. Fitted peaks of Ni^{2+}-CO (dotted green line), Ni^{2+}-$(CO)_2$ (dotted red line), the cumulative fit (dotted blue line) and experimental spectra (solid orange line) are provided. Spectra were normalized to lattice vibrations (approximately between 2100 cm^{-1} and 1700 cm^{-1}) for comparison between different samples.

Table S6. Results of peak deconvolution of normalized IR difference spectra upon low-temperature CO-adsorption (at $p_{CO} = 1 \cdot 10^{-2}$ mbar) on different Ni-NaMFI zeolite samples. CO dosing was performed at liquid N_2 temperature. Deconvolution was performed with two Gaussian peaks and a fixed baseline applying Levenberg-Marquardt iteration mechanism. Peak center, peak area, full width at half maximum (FWHM) are summarized for each peak.

Sample	Peak	Peak center /cm^{-1}	Peak area /10^{-3} counts·cm^{-1}	FWHM /cm^{-1}
Ni-NaMFI(85)	1	2206.6 ± 2.4	2.9 ± 1.4	11.4 ± 2.3
	2	2211.0 ± 0.5	1.6 ± 1.3	5.8 ± 2.0
Ni-NaMFI(179)	1	2208.1 ± 2.2	6.2 ± 2.9	11.5 ± 1.9
	2	2212.0 ± 0.4	5.0 ± 2.8	6.0 ± 1.3
Ni-NaMFI(496)	1	2207.3 ± 1.5	8.0 ± 2.0	13.1 ± 1.5
	2	2212.4 ± 0.2	7.3 ± 1.9	6.5 ± 0.7
Ni-NaMFI(671)	1	2206.4 ± 2.5	3.2 ± 1.5	11.5 ± 2.6
	2	2211.7 ± 0.2	3.0 ± 1.4	6.2 ± 1.0

S4. Physicochemical Characterization of FAU Zeolites

Table S7. Na, Ni, BAS and LAS concentration in NaFAU and Ni-NaFAU zeolite samples with varying Ni loadings.

**Sample**	**Na** **/$\mu mol_{Na}\cdot g_{zeolite}^{-1}$**	**Ni** **/$\mu mol_{Ni}\cdot g_{zeolite}^{-1}$**	**BAS** **/$\mu mol_{BAS}\cdot g_{zeolite}^{-1}$**	**LAS** **/$\mu mol_{LAS}\cdot g_{zeolite}^{-1}$**
NaFAU	1217	-	8	810
Ni-NaFAU(47)	700	47	89	190
Ni-NaFAU(50)	800	50	130	219
Ni-NaFAU(114)	731	114	62	116
Ni-NaFAU(199)	735	199	28	20
Ni-NaFAU(295)	766	295	24	26
Ni-NaFAU(607)	770	607	89	118

Table S8. Na, Ni, BAS and LAS concentrations in HFAU and Ni-HFAU zeolite samples with varying Ni loadings.

**Sample**	**Na** **/$\mu mol_{Na} \cdot g_{zeolite}^{-1}$**	**Ni** **/$\mu mol_{Ni} \cdot g_{zeolite}^{-1}$**	**BAS** **/$\mu mol_{BAS} \cdot g_{zeolite}^{-1}$**	**LAS** **/$\mu mol_{LAS} \cdot g_{zeolite}^{-1}$**
HFAU	-	-	430	168
Ni-HFAU(29)	8	29	419	295
Ni-HFAU(55)	10	55	204	151
Ni-HFAU(116)	10	116	295	275
Ni-HFAU(220)	8	220	186	497
Ni-HFAU(295)	10	295	207	446

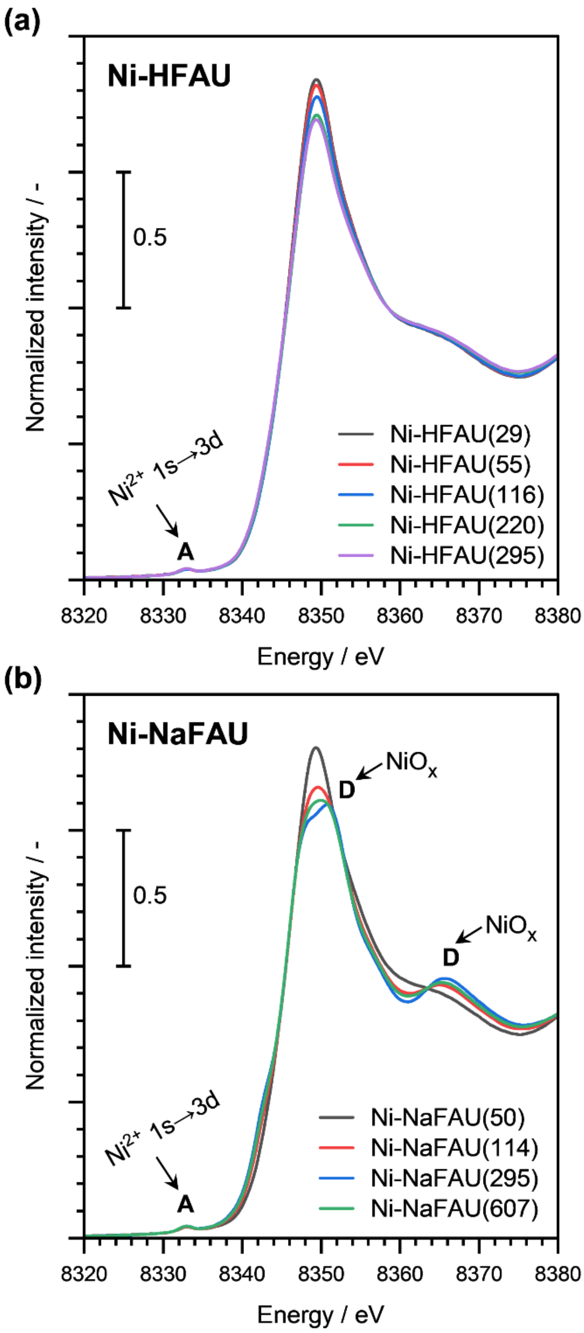

Figure S7. Ni K-edge XANES of as-synthesized Ni-NaFAU (top panel) and Ni-HFAU (bottom panel) zeolite samples with different Ni loadings. The pre-edge feature labeled as "A" corresponds to Ni^{2+} 1s → 3d electronic transitions, while the features labeled as "D" correspond to the presence of bulk NiO nanoparticles.

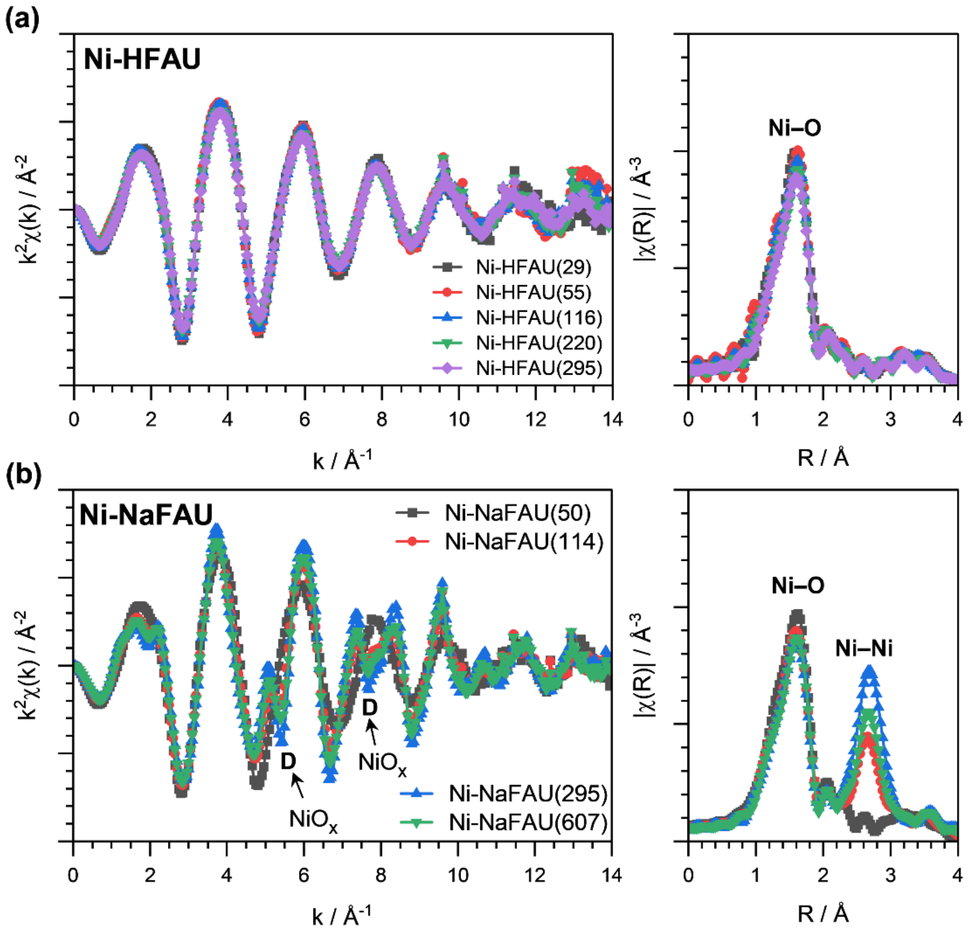

Figure S8. Ni K-edge k^2-weighted EXAFS (left panels) and FT-EXAFS (right panels) of the as-synthesized (a) Ni-HFAU and (b) Ni-NaFAU zeolite samples with varying Ni loadings. The features labeled as “D” correspond to the presence of bulk NiO_x nanoparticles.

S5. Additional Figures

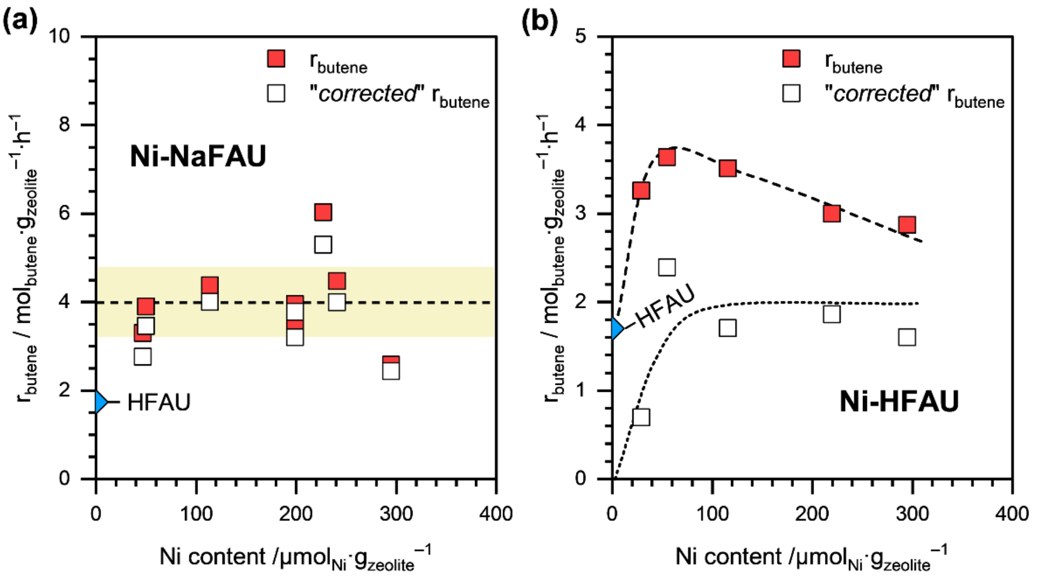

Figure S9. 1-Butene consumption rates, normalized to the catalyst mass, as a function of Ni content in (a) Ni-NaFAU and (b) Ni-HFAU zeolite samples with varying Ni loadings (red squares). The 1-butene consumption rate on the parent HFAU zeolite (blue diamond) is also shown for comparison. The "corrected" 1-butene consumption rates, after subtracting the contribution from the residual Brønsted acid sites are presented as open squares.

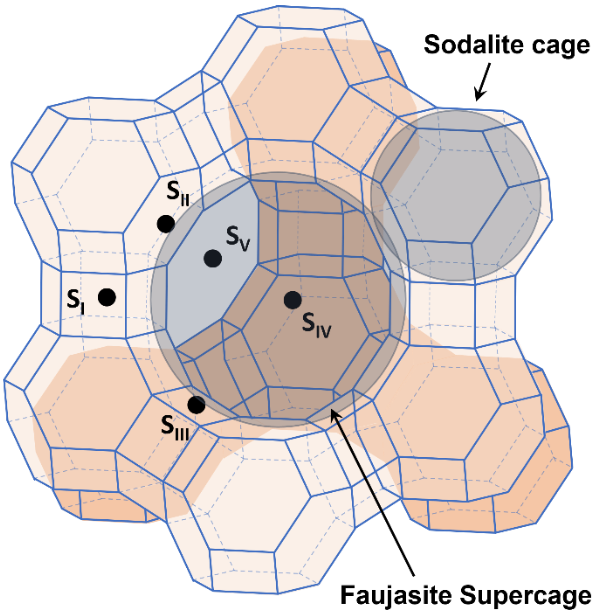

Figure S10. Different locations of mono- and di- cations in the FAU framework. S_I: in the center of the hexagonal prism. S_{II}: in the center six-membered ring between the sodalite cage and the faujasite supercage. S_{III}: between the hexagonal prism and the faujasite supercage. S_{IV}: in the middle of the faujasite supercage. S_V: between the two faujasite supercages.

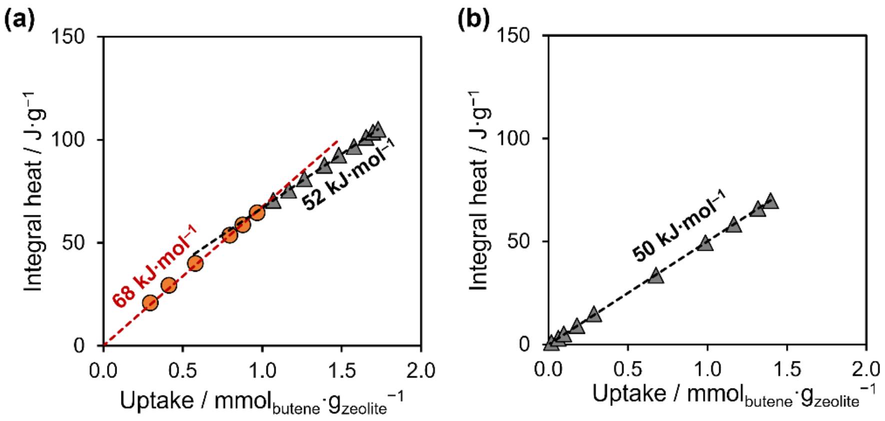

Figure S11. Integral heats as a function of butene uptake on (a) Ni-NaMFI(85) zeolite samples and (b) silicalite. The integral heat of Ni-NaMFI shows two contributions: the adsorption on Ni and Na LAS (orange circles) and the physisorption within the zeolite pores resembling the interaction with the zeolite framework (gray triangles).

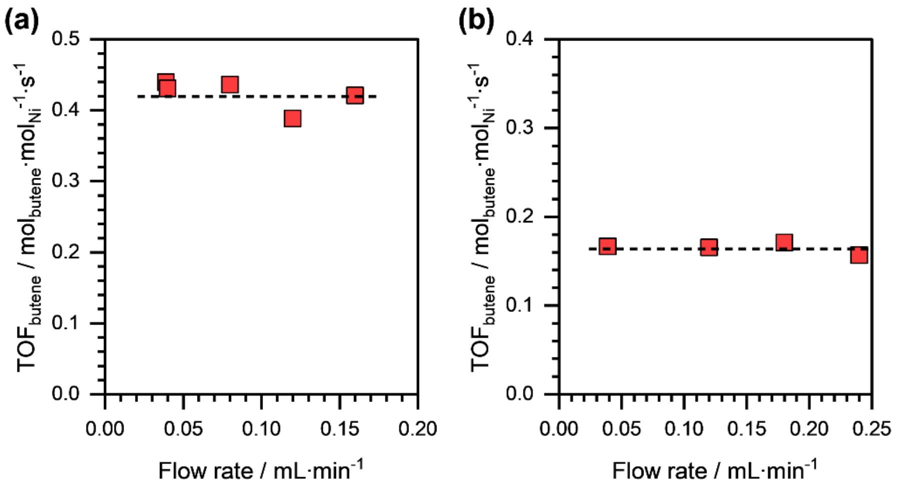

Figure S12. 1-Butene conversion turn-over frequency (TOF_{butene}) as a function of butene flow rate (at constant space-velocity achieved by varying the catalyst mass) on representative Ni-NaFAU (left panel) and Ni-NaCHA (right panel) catalyst samples. Reaction conditions: T ≈ 433 K, p_{total} ≈ 50 bar (15% isobutane and 85% 1-butene), space-velocity ≈ 4.5 mmol$_{butene}$·g$_{zeolite}$$^{-1}$·s^{-1} for Ni-NaFAU and ~0.36 mmol$_{butene}$·g$_{zeolite}$$^{-1}$·s^{-1} for Ni-NaCHA. All reactions were performed under differential (<10%) conversion conditions.

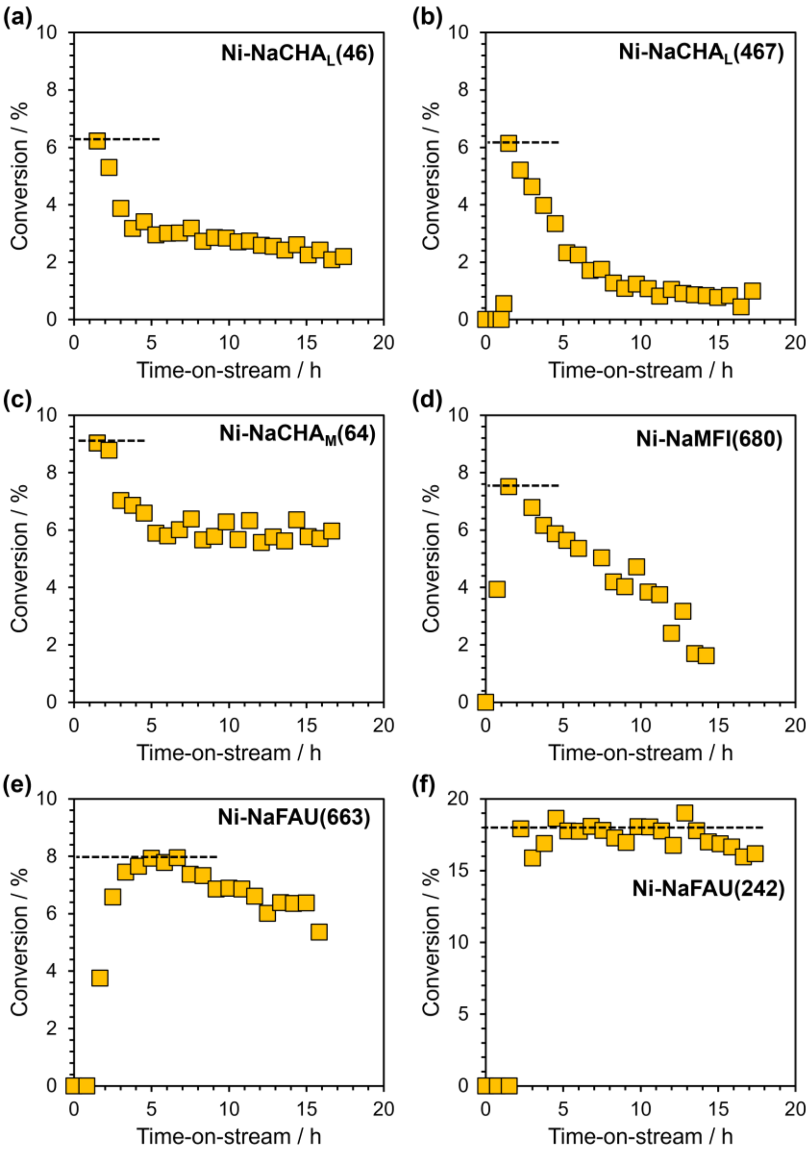

Figure S13. Exemplary 1-butene conversion *versus* time-on-stream (TOS) plots for representative Ni-NaCHA, Ni-NaMFI and Ni-NaMFAU zeolite samples. Reaction conditions: $T \approx 433$ K, $p_{total} \approx 50$ bar (15% isobutane and 85% 1-butene). The dashed lines represent the conversion value that was used to estimate the 1-butene conversion rates for these catalysts.

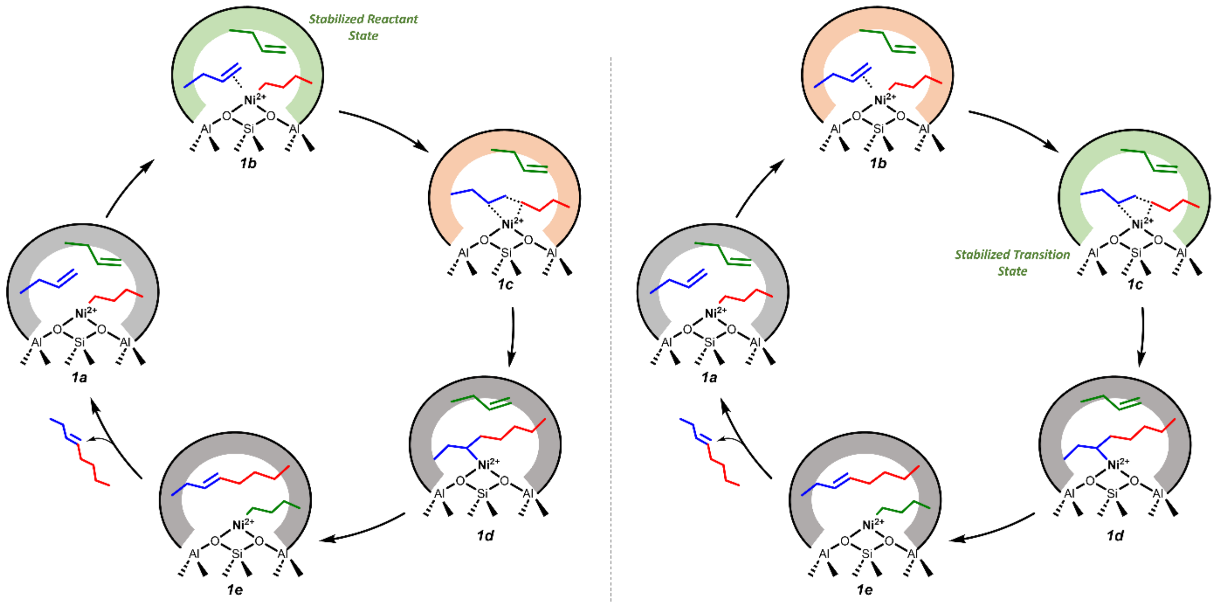

Scheme S1. A schematic illustration of the differential stabilization of the initial state and the final state in small-pore zeolites like CHA and MFI (left panel), and in large-pore zeolite like FAU (right panel).

References

[1] J.R. Di Iorio, R. Gounder, Controlling the Isolation and Pairing of Aluminum in Chabazite Zeolites Using Mixtures of Organic and Inorganic Structure-Directing Agents, Chemistry of Materials, 28 (2016) 2236-2247.

[2] U. Deka, A. Juhin, E.A. Eilertsen, H. Emerich, M.A. Green, S.T. Korhonen, B.M. Weckhuysen, A.M. Beale, Confirmation of Isolated Cu2+ Ions in SSZ-13 Zeolite as Active Sites in NH3-Selective Catalytic Reduction, The Journal of Physical Chemistry C, 116 (2012) 4809-4818.

[3] E. Welter, R. Chernikov, M. Herrmann, R. Nemausat, A beamline for bulk sample x-ray absorption spectroscopy at the high brilliance storage ring PETRA III, AIP Conference Proceedings, 2054 (2019).

[4] B. Ravel, M. Newville, ATHENA, ARTEMIS, HEPHAESTUS: data analysis for X-ray absorption spectroscopy using IFEFFIT, Journal of Synchrotron Radiation, 12 (2005) 537-541.

[5] K. Hadjiivanov, H. Knözinger, M. Mihaylov, FTIR study of CO adsorption on Ni-ZSM-5, The Journal of Physical Chemistry B, 106 (2002) 2618-2624.

[6] S. Moussa, P. Concepción, M.A. Arribas, A. Martínez, Nature of active nickel sites and initiation mechanism for ethylene oligomerization on heterogeneous Ni-beta catalysts, ACS Catalysis, 8 (2018) 3903-3912.

[7] M. Kermarec, D. Olivier, M. Richard, M. Che, F. Bozon-Verduraz, Electron paramagnetic resonance and infrared studies of the genesis and reactivity toward carbon monoxide of Ni^{+} ions in a NiCa-X zeolite, The Journal of Physical Chemistry, 86 (1982) 2818-2827.

[8] S.M.T. Almutairi, B. Mezari, E.A. Pidko, P.C.M.M. Magusin, E.J.M. Hensen, Influence of steaming on the acidity and the methanol conversion reaction of HZSM-5 zeolite, Journal of Catalysis, 307 (2013) 194-203.

[9] A. Zecchina, S. Bordiga, C. Lamberti, G. Spoto, L. Carnelli, C. Otero Arean, Low-temperature fourier transform infrared study of the interaction of CO with cations in alkali-metal exchanged ZSM-5 zeolites, The Journal of Physical Chemistry, 98 (1994) 9577-9582.

[10] K. Hadjiivanov, M. Mihaylov, D. Klissurski, P. Stefanov, N. Abadjieva, E. Vassileva, L. Mintchev, Characterization of Ni/SiO_2 catalysts prepared by successive deposition and reduction of Ni^{2+} Ions, Journal of Catalysis, 185 (1999) 314-323.
